# Supplementary material for: Position: A study protocol for the prevention of fall injuries in french special forces selection courses using a body-centered intervention
Source: PLoS One. 2023 Oct 4;18(10):e0290241. doi: 10.1371/journal.pone.0290241 (PMC10550174; doi:10.1371/journal.pone.0290241)
Supplement: S1 File — (DOCX) [file pone.0290241.s001.docx]

**Sessions - Active Control Group**

| **Session number** | **Main idea** | **Cognitive process** | **Activity/ activities** | **Organization of the session**  **(duration, group)** |
| --- | --- | --- | --- | --- |
| 1 | Presentation and identification of the different sensory channels | Perception and attention | Presentation of VAKOG  (Visual, Auditory, Olfactory and Gustatory)  Identification of the main sensory channel  Example applications | 45–60 minutes  In groups of 20/25 people maximum  Practical exercises in groups of 5 to 10 participants, depending on the activity |
|  | Integration of information | Memory /  Working memory | Presentation of memory processes, with a focus on working memory  Example of an optimization tool: the Memory Palace  Practical exercises working in pairs and groups |  |
| 3 | Reasoning 1 | Reasoning, coping strategy | Presentation of the different types of reasoning  Individual and group ‘psycho-technical’-type exercises |  |
| 4 | Reasoning 2 | Conceptualization, expression and communication of ideas | Dissertation topics: immortality, new technologies in the military, self-sacrifice, emotions and taking action, etc. |  |
| 5 | Mental procedure before taking action | Mental imaging | Presentation of mental imagery, its use in the professional context and performance objectives (example of the PAF and high-level athletes)  Individual exercises (the tower of Hanoi), then as a group (the marshmallow challenge) |  |
| 6 | Faced with a problem, using imagination and creativity as a solution | Imagination, divergent and convergent thinking | Presentation of the theme ‘creativity and active thinking’  Individual problem-solving exercises and inter-group challenges |  |

* Divergent thinking: producing many ideas from one stimulus. Convergent thinking: producing a single solution that integrates several elements.

**Sessions - ORAF Group**

| **Session number** | **Theme** | **Techniques worked on** | **Content** | **Organization of the session**  **(duration, group)** |
| --- | --- | --- | --- | --- |
| 1 | ORAF Presentation - Breathing | Breathing - Relaxation | Presentation of the ORAF: philosophy, theory & application  Breathing: theory and practice | 45 to 60 minutes  In groups of up to 20 people |
| 2 | Sleep and Vigilance | Relaxing breathing and calm imagery | Sleep/Vigilance/Fatigue theory  Continual awareness of the whole body through relaxation (recovery and relaxation); using VAKOG to support an internal dialogue/relaxing imagery |  |
| 3 | Stress | The optimized activation signal (OAS) technique | Theoretical presentation of stress  Implementation of the OAS technique |  |
| 4 | Self-confidence, performance optimization | Relaxing breathing  Mental rehearsal | Mental rehearsal: presentation and practical exercise |  |
| 5 | Mindfulness and performance optimization | Geolocation  (taking the environment into account)  Dynamic breathing | Practical geolocation exercise, optimized complete dynamization and immediate mental projection |  |
| 6 | Motivation and performance | Relaxation  Mental projection of goals (MPG) | Motivation theory + MPG  Individual relaxation exercise, followed by an MPG exercise |  |
