# Supplementary material for: Position: A study protocol for the prevention of fall injuries in french special forces selection courses using a body-centered intervention
Source: PLoS One. 2023 Oct 4;18(10):e0290241. doi: 10.1371/journal.pone.0290241 (PMC10550174; doi:10.1371/journal.pone.0290241)
Supplement: S3 File — (DOCX) [file pone.0290241.s003.docx]

**RIPH2 PROTOCOL**

**PréventiOn de la bleSsure en milieu militaIre**

**par opTImisation de la cONscience corporelle**

**POSITION**

**(Preventing injury in the military environment**

**by optimizing body consciousness)**

***

Version 1.0 of 07/21/2021

Sponsor reference: 2020PBMD04 - IDRCB reference: 2021-A02108-33

# LIST OF ABBREVIATIONS

ANSM Agence nationale de sécurité du médicament et des produits de santé
(National Agency for the Safety of Medicines and Health Products, equivalent to the FDA)

BESS Balance Error Scoring System

CNIL Commission nationale de l’informatique et des libertés (French Data Protection Authority)

CRF Case report form

CV Curriculum vitae

DPO Data Protection Officer

EPMS Entrainement physique militaire et sportif (Military and sports physical training)

GCP Good clinical practice

IRB Institutional Review Board (Comité de Protection des Personnes)

IRBA Army Biomedical Research Institute (Institut de recherche biomédicale des armées)

RM Reference methodology

RPPS Shared directory of healthcare professionals (Répertoire partagé des professionnels de santé)

SSA Service de santé des armées (Armed Forces Health Service)

STAC Stage de sélection des commandos marines (Marine commando selection course)

POT Potential optimization techniques

# RESEARCH SUMMARY

| Sponsor | Direction Centrale du Service de Santé des Armées |
| --- | --- |
| Person who will direct and supervise the study | Anaïs DUFFAUD, PhD  Unité neurophysiologie du stress, Département neurosciences et sciences cognitives, Institut de Recherche Biomédicale des Armées, BP 73, 91223 Brétigny-sur-Orge, France |
| Title | Preventing injury in the military by optimizing body awareness - POSITION |
| Justification / context | Physical activity and sport play an important role in the military environment, and help to develop and optimize the operational capacity of combatants. Nevertheless, the injury rate is high, and the identification of risk factors that are amenable to primary prevention measures is a key challenge in the context of maintaining combatants in good health. Military epidemiological data show that the majority of injuries are acute trauma due to a fall following a loss of balance. These data suggest that postural balance may moderate the occurrence of injuries that occur during physical activities in this context. Furthermore, the high stress levels that individuals are exposed to during their military physical activities could contribute to a degradation in postural balance. The POSITION project investigates the cognitive mechanisms involved in postural awareness as a risk factor for injury, and as a target for a primary prevention strategy based on Potential Optimization Techniques (POTs)^[[1]](#footnote-1)^. POTs could improve postural control, and reduce the intensity of the psychobiological stress response, thereby reducing the rate of fall-related injuries that occur during physical activities in the military context. |
| Objectives | The main objective is to evaluate the effectiveness of POTs in preventing fall injuries during physical activities in the military context.  Secondary objectives:  The first secondary objective is to test the mechanistic hypothesis that POTs can reduce the risk of fall injury by improving postural awareness.  The second secondary objective is to test the mechanistic hypothesis that POTs can reduce the risk of fall injury by decreasing the intensity of the psychobiological stress response during physical activities in the military context.  The third secondary objective is to use machine learning to develop a predictive model of the risk of fall injury, based on clinical (the BESS test) and paraclinical (posturography) measurements taken prior to the start of physical activities in the military context.  The fourth secondary objective is to evaluate the persistence over time of the effectiveness of a POT intervention in preventing the risk of fall-related injury. The rate of fall injuries will be compared for POTs and active control groups among participants who continue to engage in physical activities after the end-of-study visit (and complete the selection course). |
| Research outline | This is an open, prospective, controlled, single-center cohort study. |
| Evaluation criteria | The primary endpoint is the rate of fall-related injuries due to a loss of balance in each of the two groups (POTs *vs*. active control).  Secondary endpoints:  The first secondary objective comprises two evaluation criteria: 1) self-reported scores on the Postural Awareness Scale pre- and post-intervention, and at the start of each session; 2) variation in measures of postural balance pre- and post-intervention.  The second objective also comprises two criteria: 1) self-reported scores on Cohen’s Perceived Stress questionnaire pre- and post-intervention, and at the start of each session; 2) variation in measures of allostatic load in saliva pre- and post-intervention (corticotropic axis: cortisol, DHEA, oxidative stress; the autonomic nervous system: alpha amylase and chromogranin A).  The evaluation criterion for the third secondary objective will be the correct prediction of the risk of fall injury by the machine learning model.  The evaluation criterion for the fourth secondary objective will be the rate of fall-related injuries following a loss of balance in each of the two groups (POTs *vs*. active control), between the end of the study and the end of the selection course. |
| Inclusion criteria | Informed written consent to participate in the study  Candidate for the selection course  Male or female, over 18 years of age  Eligible for benefits under the French Social Security scheme |
| Exclusion criteria | Participants who have already completed an ‘advanced’ POTs training program (> 10 hours)  Refusal to participate  Person covered by articles L1121-5 to L1121-8 of the French public health code, as follows:   - A pregnant, parturient or nursing woman, - A person detained following a judicial or administrative decision, - Persons who are subject to psychiatric monitoring under articles L3112-1 and L3113-1, and who are not covered by the provisions of article L1121-8, - Adults made a ward of court, or who are unable to give their consent. |
| Schedule | The study will consist of the following visits:   - 1 inclusion visit: inclusion interview and written consent - 1 initial assessment visit: self-report questionnaires (medico-biographical, postural awareness (Postural Awareness Scale) and stress (Cohen’s Perceived Stress Scale), clinical and paraclinical posture measurements and saliva collection. - 4 follow-up visits carried out at the start of the POT or active control sessions, consisting of: self-report questionnaires (postural awareness and perceived stress), - there will be 1 additional visit for participants who suffer a fall-related trauma during the course. The participant will complete questionnaires measuring postural awareness and perceived stress, and a posturography measurement will be performed. - 1 end-of-study visit will take place on the day following the last session of the POT or the active control, or on the day of the fall injury if it occurs before the end of the POT or active control intervention. This visit will include: paraclinical measurement of posture, self-report questionnaires (postural awareness and perceived stress) and saliva collection. |
| Number of subjects | The sample should include 200 participants in each of the two groups (POT and active control), making a total of 400 participants. |
| Statistical analysis | For the primary objective, the effect of the intervention (POT *vs.* active control) on the rate of fall-related injuries will be evaluated using a comparative survival analysis (the Cox model, with clinical variables as potential confounders).  For the first secondary objective, the effect of the POT intervention on postural awareness will be evaluated by comparing the following for the POT group: (*i*) scores on the Postural Awareness Scale at the six measurement times (repeated measures ANOVA), and (*ii*) posturography measurements pre- and post-intervention (paired samples comparison of means).  For the second secondary objective, the effect of the POT intervention on the psychobiological stress response will be evaluated by comparing the following measures for the POT group at six measurement times: (*i*) scores on Cohen’s Perceived Stress Questionnaire (repeated measures ANOVA), and (*ii*) biological stress variables (paired samples comparison of means).  For the third secondary objective, machine learning will be used to evaluate the accuracy of the predictive model of the risk of fall injury based on the accuracy on the prediction.  For the fourth secondary objective, the long-term effectiveness of the POT intervention in preventing fall-related injuries, between the end of the study and the end of the selection course will be evaluated using a comparative survival analysis (the Cox model). |
| Study duration | Duration of inclusion: 24 months  Duration of the intervention: 3 weeks  Duration of each participant’s participation: 4 weeks  Total study time: 25 months |

# SCIENTIFIC JUSTIFICATION

## Current knowledge

### Pathology/ health problems and current knowledge

Physical activity and sport play an important role in the military, as they are used to develop and optimize the operational capacity of combatants. In France, *Entraînement Physique Militaire et Sportif* (Physical and Sport Training for the Military) is designed to enable all military personnel to acquire the physical and mental capacities they need to perform their duties (Etat-major des armées - Centre national des sports de la défense, 2011). In addition, the preservation of operational capacity throughout a military career is based, among other things, on the health benefits of sporting activities, notably with respect to the prevention of chronic, stress-related pathologies such as cardiovascular disease and depression (World Health Organization, 2003).

Nevertheless, physical activity and sport can also be the source of acute (sprains, fractures, etc.) and chronic (tendinopathies) musculoskeletal pathologies. In particular, there is a high rate of lower-limb trauma during physical activities in the military context, both among young enlisted personnel [15–20%] (At, 2016; Bauvent, 2014; Pleche, 2018) in operational military units or during selection courses for specialized units [30–45%] (Bertrand, 2016; Brocard, 2014; Chipault, 2016; Longin, 2015; Morinière, 2013). In addition to their impact on the health of combatants, these injuries have a negative impact on the operational capability of forces (due to incapacity) and a significant economic cost (Ressort, Desjeux, Marsan, & Thevenin-Garron, 2013)

Identifying risk factors is an essential step in developing an injury prevention strategy. Classically, there are two categories of risk factors: (*i*) those that are intrinsic to the individual, and include age, gender, physical condition or tobacco consumption; and (*ii*) extrinsic factors, which relate to the context in which the sporting activity is performed, and which include environmental conditions (temperature, humidity, etc.), the type of activity, or training conditions (progression, regularity, etc.). (Bigard, Cravic, & Banzet, 2010; Meeuwisse, 1994; Williams, 1971). The identification of modifiable risk factors (i.e. those that are amenable to an intervention that will reduce the likelihood of injury), remains a major challenge in the field of sport (Bahr & Holme, 2003).

Against this background, the POSITION project aims to study the cognitive mechanisms involved in body awareness, both as a risk factor in fall injury, and as a target for a prevention strategy that aims to reduce the injury rate by improving postural control.

This research project was prompted by epidemiological observations carried out during the French Marine Commando Selection Course (STAC). Medical unfitness due to incapacity is the leading cause of failure (70%). Acute trauma is the principal pathology (60%), typically involving the lower limbs (Bertrand, 2016; Longin, 2015; Morinière, 2013). A review of the circumstances surrounding the trauma highlighted that a fall due to a loss of balance was the cause in 90% of cases (Bertrand, 2016). These data suggest that postural balance could play a role in the injuries encountered during the STAC. The activities that candidates must undertake require them to have extremely good postural balance: trainees carry heavy loads (a combat bag and a weapon, weighing 15 kg) for prolonged periods, and complete extreme challenges that are run at-height in all weather conditions. Epidemiological data collected during the course show that the leading activity resulting in trauma is the ‘jungle’ challenge, which involves overcoming obstacles up to 5 meters high with no safety net (Morinière, 2013). Moreover, preliminary (unpublished) epidemiological data collected from other military units (the National Gendarmerie, the 1st Marine Infantry Parachute Regiment, the 13th Parachute Dragoon Regiment, the 10th Air Parachute Commando, and the Fusiliers Marins [Navy Riflemen] School, Paris Fire Brigade) seem to confirm the high rate of trauma linked to a loss of postural balance during physical activities.

It should be noted that the physical activities carried out by the aforementioned military units are characterized by a very high level of physical and psychological demands, which challenge the individual’s physical capacities and exposes them to a high level of stress. The individual’s limits are pushed to the extreme by demanding and intense physical exercises (each exercise being carried out in combat gear). Another challenge is the constant psychological testing (ongoing stressors such as a lack of sleep, uncertainty about what the day will hold, etc.), which ensure that only the best candidates are selected for the missions undertaken by elite military units (Bertrand, 2016). Together, these challenges activate the neurobiological stress response. The latter, aspecific biological mechanism is a response to both psychological or physical stress; it enables the organism to cope with changes in its environment that affect its integrity, and to adapt if these changes persist. It depends not only on the characteristics of the stressor, but also on the subject’s general psychobiological response.

### Current knowledge of the issues addressed by the protocol

Several neurophysiological systems contribute to postural balance, which is more than the reflexive action of the postural muscles. Postural balance involves various high-level cognitive processes such as attention, visuo-spatial perception and executive functions (Amboni, Barone, & Hausdorff, 2013). Sensory information from peripheral receptors (located in the visual, vestibular and somatosensory systems) is integrated by the central nervous system, which, in turn, controls the muscles that orient the various parts of the body with respect to gravity (Forbes, Chen, & Blouin, 2018). The cognitive mechanisms involved in postural awareness refer to the attention that the individual pays to information from somatosensory and vestibular systems (Mehling et al., 2009). The more attention that the individual pays to his or her body, the more information is available to them to control their posture. In other words, it is possible that postural control may be enhanced by a high level of body awareness. In the context of the POSITION project, we hypothesize that the individual level of postural awareness could modulate the risk of fall-related trauma. In particular, we propose that a low level of awareness could be a risk factor for fall-related trauma, *via* impaired postural control. Furthermore, we hypothesize that improving postural control, by increasing the level of postural awareness, could be a primary prevention strategy.

It should be noted that the neurophysiological systems involved in postural control are influenced by brain structures that are not part of the postural control loop. For example, the degree of excitability of the postural myotatic reflex at the level of the spinal cord is modulated by subcortical structures (notably the periaqueductal gray matter and the amygdala) that are activated during the stress response (Volchan et al., 2017). Interestingly, body awareness plays an important role in regulating the stress response by contributing to the identification, evaluation and regulation of the body’s internal physiological state that characterizes the neurobiological stress response (Craig, 2002). These observations suggest that postural balance may be indirectly modulated by cognitive mechanisms governing body awareness, *via* control of non-postural responses (e.g., the stress response) that influence posture. In sum, the body awareness mechanism could modulate the occurrence of fall injuries either: (*i*) directly as a central actor in the postural control loop; or (*ii*) indirectly by acting on non-postural pathways that affect posture. Figure 1 summarizes the theoretical model we will be testing in the context of the POSITION project.


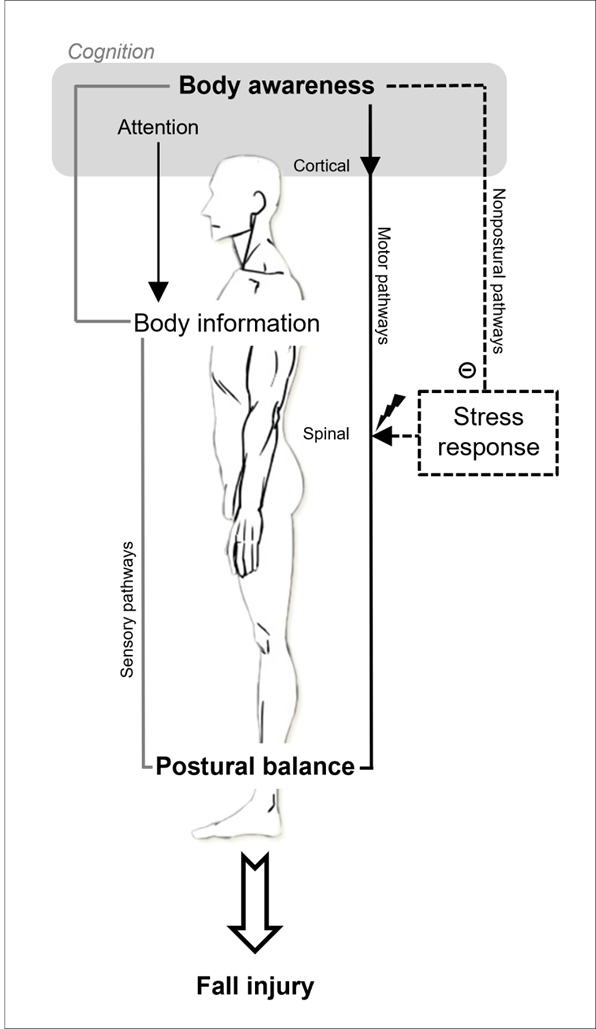


**Figure 1** – The theoretical model according to which cognitive mechanisms governing body awareness could modulate the occurrence of fall injuries, either: *(i)* directly by acting on descending motor pathways (black arrow) as body awareness is a central process in the postural control loop (solid black lines); or *(ii)* indirectly by controlling non-postural pathways (grey arrow) such as the stress response which alters postural control.

Classically, quiet standing is described by the biomechanical ‘inverted

Classically, ‘quiet standing’ is described by the biomechanical ‘inverted pendulum’ model (Gage, Winter, Frank, & Adkin, 2004): the body moves continuously along an antero-posterior axis and a medio-lateral axis, both of which originate in the medial malleoli of the ankles (Figure 2). The standing position is naturally unstable due to the physiognomy of the human body (⅔ of body mass is located in the upper ⅔ of body height), and endogenous physiological disturbances (breathing, heartbeats, etc.). Postural control is therefore continuously deployed to maintain the body around a point of equilibrium, and consequently avoid a fall.


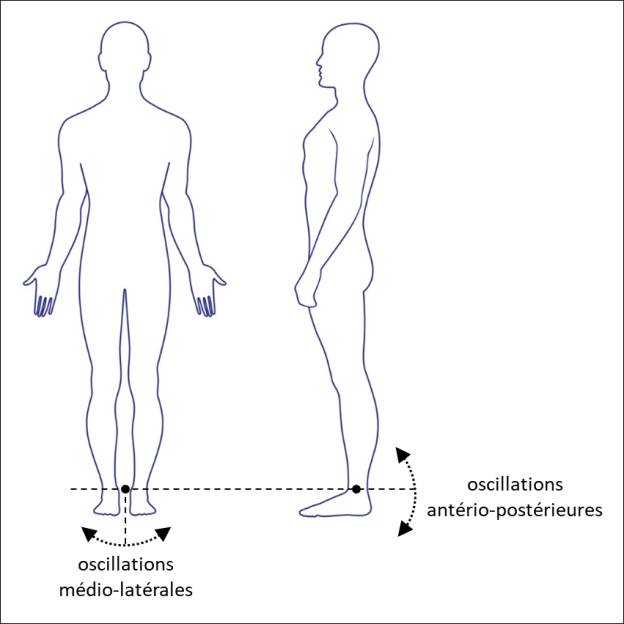


medio-lateral

oscillations

antero-posterior

oscillations

**Figure 2 –** Biomechanical model of static postural balance, also known as the ‘inverted pendulum’ model. The body moves continuously along an antero-posterior axis and a medio-lateral axis, both originating in the medial malleoli of the ankles.

In the context of sports medicine, static postural balance is most often assessed by the Balance Error Scoring System (BESS) (Delahunt et al., 2018; Docherty, Valovich McLeod, & Shultz, 2006). This clinical test must be performed by a physician, and lasts around five minutes. In the field of cognitive neuroscience, recent work has shown the value of a paraclinical tool, posturography, to assess postural balance and investigate the individual’s level of body awareness (C. Verdonk, Trousselard, Medani, Vialatte, & Dreyfus, 2020). The analytical tools developed in the context of the latter study were subsequently used to develop another tool to assess the risk of fall injury during the STAC. Preliminary analyses show that posturography was able to correctly assess the risk of injury in 75% of trainee marine commandos (C. Verdonk et al., 2019).

The POSITION project focuses on the role of cognitive mechanisms governing body awareness as a risk factor for fall injuries during physical activities in the military. In this context, there is a clear need to evaluate the modifiability of the latter risk factor in order to develop a prevention strategy. ‘Modifiability’ means that the risk factor is amenable to an intervention that will modulate its influence on the occurrence of injury (Bahr & Holme, 2003). Specifically, the project will test the effect of *Potential Optimization Techniques* (POTs) on the cognitive mechanisms governing body awareness. We hypothesize that POTs will increase body awareness, and thus reduce the risk of fall-related injury, *via* improved postural control.

POTs were created in the 1990s by Doctor Edith Perreault-Pierre to meet needs that had never before been addressed in the armed forces: mental preparation and recovery. (Etat-major des armées - Centre national des sports de la défense, 2011). Initially developed to meet the requirements of the French Air Force regarding stress management and human factors (in the context of flight safety), they are now being deployed throughout the French Army’s other corps.

POTs are defined as a preparation method that is focused on learning a set of mental techniques and strategies that will help the individual to use their physical and psychological resources as effectively as possible, as a function of the demands of the situation they find themselves in (Figure 3).

**Techniques**:

A set of resources or tools that support the best performance possible

**Potential**:

The strengths, capacities, abilities and resources available to the subject.

**Optimize**:

Help in motivating the individual to perform as well as possible as a function of the demands of the situation

**POT**

Context

Context

Context

**Figure 3 –** The 3 components making up Potential Optimization Techniques (POTs).

The techniques draw upon basic procedures found in high-level sport. The method takes the form of a customizable ‘toolbox’ and the aim is to enable trained personnel to complete their missions successfully, while preventing the deleterious effects of stress. On the one hand, the positive effects of mental imagery, internal dialogue, relaxation and mindfulness techniques on performance, health and well-being are well documented in the literature (Pagninia, Manzonib, Castelnuovoab, & Molinariab, 2013; Vealey & Greenleaf, 2010; William, Zinsser, & Bunker, 2010). On the other hand, evidence regarding the effectiveness of POTs is inconclusive. The Table included in APPENDIX 1 summarizes studies that have evaluated the effects of POT training on psycho-cognitive and physical performance, stress and sleep. Although the quality of these studies varies (several before-and-after interventional studies, and one randomized controlled trial), taken together, the results support the clinical and operational benefits of POTs.

## Research hypotheses

The POSITION project aims to evaluate the effectiveness of POTs in preventing fall injuries following a loss of balance^[[2]](#footnote-2)^. We hypothesize that POTs could reduce the risk of fall injury by improving postural control through improved postural awareness.

## Expected benefits

In the military context, physical activities are associated with a high rate of fall-related injuries. Consequently, a key challenge for military medics is to identify individuals who are at-risk, in order to be able to offer them personalized preventive countermeasures to reduce their risk. There is a need to develop a tool that can assess risk factors that are amenable to a prevention strategy that reduces the risk of injury. One of the outcomes of the POSITION project will be the development of a tool to identify individuals at-risk of fall injury, based on a postural balance analysis (assessed using the BESS and posturography).

In addition, the investigation of the cognitive mechanisms involved in postural awareness will provide a better understanding of inter-individual variability regarding fall injury risk during physical activities in the military context. Finally, an assessment of the ability of POTs to reduce the risk of injury will support the evaluation of the program, which is already used by the French armed forces, in preventing fall-related trauma.

# OBJECTIVES

## Main objective

The main objective is to evaluate the effectiveness of POTs in preventing fall injuries during physical activities in the military context. To this end, we will analyze the effect of a POT intervention on the rate of fall-related injuries that occur during physical activities in a military population, compared to an active control.

## Secondary objectives

The study has 4 secondary objectives:

1. The first is to test the mechanistic hypothesis that POTs reduce the risk of fall-related injury by improving postural awareness.
2. The second is to test the mechanistic hypothesis that POTs reduce the risk of fall injury by decreasing the intensity of the psychobiological stress response during physical activities in the military context.
3. The third is to use machine learning techniques to develop a predictive model of the risk of fall injury, based on a clinical test (BESS) and paraclinical (posturography) measurements taken before physical activities begin.
4. The fourth is to evaluate the persistence over time of the effectiveness of a POT intervention in reducing the risk of fall injury. The assessment will be based on participants who continue to engage in physical activities after the end-of-study visit (and who complete the selection course); the analysis will compare the rate of fall injuries for POTs and active control groups.

# STUDY OUTLINE

## Type of experiment

This study falls within the scope of interventional research that studies the human body, with minimal risks and constraints. This is due to the fact that, on the one hand it evaluates a POTs intervention, which is already being used by the French Ministry of the Armed Forces, and, on the other hand, it requires the collection of non-invasive biomedical (physiological and psychological) data, which provide no information regarding the individual’s health.

The project’s objectives will be met using a prospective, controlled cohort study. This open study will be conducted in a sample of healthy volunteers, with direct benefits. It is one of various activities typically implemented during physical activities in the military context to ensure that personnel remain in good health.

## Study outline

In order to guarantee compliance with the experimental protocol over the two years of the study (see paragraph 3.3 for a detailed explanation), the active control intervention will be carried out in the first year (year Y), while the POTs intervention will be carried out in the second year (year Y+1).

The study will be carried out in the following six units the in the French armed forces :

1. the National Gendarmerie (Versailles-Satory, 78)
2. the 10th Air Parachute Commando (Orléans, 45)
3. the 1st Marine Infantry Parachute Regiment (Bayonne, 64)
4. the 13th Parachute Dragoon Regiment (Souge, 33)
5. the Paris Fire Brigade (Villeneuve-Saint-Georges, 94)
6. the Fusiliers Marins [Navy Riflemen] School (Lorient, 56)

The study will take place during the selection courses organized by each unit, and will last three weeks.

### The intervention

The POTs intervention will consist of practical courses run by the designated POTs instructor in each military unit. This person is a member of the military who has completed the POT trainer course delivered by the French Centre National des Sports de Défense. The intervention is divided into six, one-hour sessions, spread over three weeks, with two sessions per week. The first two sessions will be scheduled at the start of the selection course (ideally the day before it begins).

The intervention will include theoretical instruction and practical exercises in POTs that have been identified as useful in increasing body awareness (paying attention to breathing, muscle relaxation, mental imagery). The contents of the six sessions will be established and validated beforehand by POTs instructors from the six military units, under the supervision of the POTs instructor who coordinates the intervention.

### The active control

The active control will unfold in the same way as the POTs intervention: there will be six one-hour sessions, spread over the three weeks of the study, with two sessions per week. These sessions will consist of exercises (details of the sessions are available in APPENDIX 2 ) that focus on high-level cognitive processes such as reasoning, information processing and creativity. They will be divided into two p3arts: the first, theoretical component will describe the processes that will be the focus of the session, and the second, practical component will focus on exercises.

These sessions will be carried out under the supervision of the person responsible for the POTs intervention that will take place the following year (the POTs instructor).

### Summary


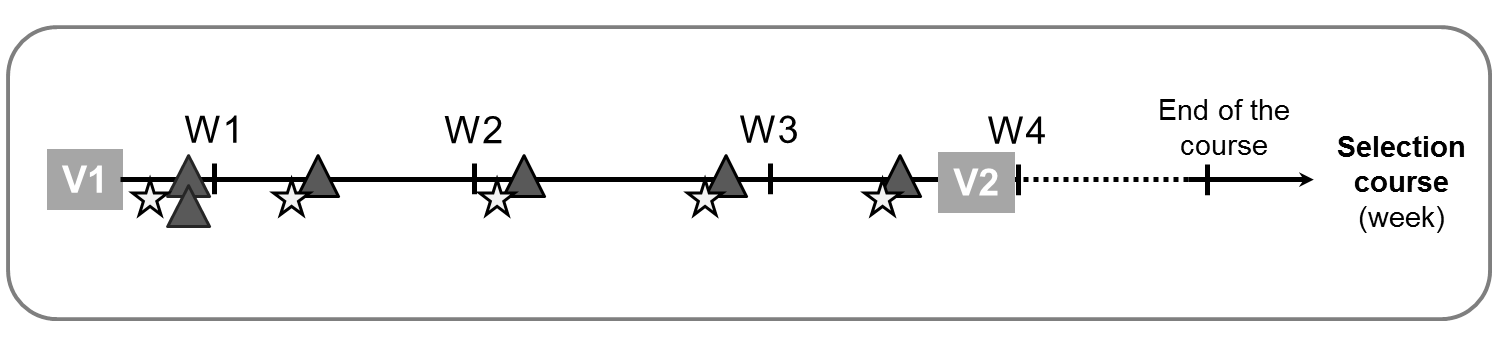


**Figure 4** – Schedule of the study for a given year. The same schedule will be followed during the two years of the study, only the content of the sessions will change: the active control in year Y, and the POTs intervention in year Y+1. Timeline for each study participant (in weeks). V1: inclusion visit (saliva sampling, posturography, and the BESS). V2: end-of-study visit (saliva sampling, and posturography). Gray triangle: a one-hour session, either during the active control program (first year), or the ORAF intervention (second year). Star: Self-administeed questionnaires (PAS, PSS, fatigue).

## Randomization

There will be no randomization (POTs versus active control) at the individual level (i.e., for each participant in each military unit). This is not possible due to the risk of participants in the POTs group influencing those in the active control group (contamination bias). The conditions under which the selection course is carried out mean that participants in both groups are in close contact with each other. Moreover, it is possible that running the two interventions at the same time may undermine the desired team cohesion. Hence, we have chosen to carry out the study over two successive years (years Y and Y+1):

1. Participants included in year Y will follow the active control program.
2. Participants included in year Y+1 will follow the POTs program.

The order of the two interventions (active control followed by POTs) is deliberately not randomized between units, to ensure that the research protocol is respected throughout the study. If the POTs intervention was run in year Y, and was found to be beneficial, it would be very difficult to expect participating units to set up an active control in year Y+1, as it is unrealistic to expect them to abandon a program with potential benefits for candidates.

# ELIGIBILITY CRITERIA

## Recruitment procedures

Participants will be volunteers recruited from among candidates for the selection course organized by the various units. They will be in good health and physical condition, as they will have been declared medically fit to enroll in the course. Participation in the study will be open to all candidates who qualify for the selection course.

## Inclusion criteria

- Informed written consent to participate in the study
- Candidate for the selection course
- Male or female, over 18 years of age
- Eligible for benefits under the French Social Security scheme

## Exclusion criteria

- Participants who have already completed an ‘advanced’ POTs training program (> 10 hours)^[[3]](#footnote-3)^
- Refusal to participate
- Person covered by articles L1121-5 to L1121-8 of the public health code, as follows:
  - A pregnant^[[4]](#footnote-4)^, parturient or nursing woman,
  - A person detained following a judicial or administrative decision,
  - Persons who are subject to psychiatric monitoring under articles L3112-1 and L3113-1, and who are not covered by the provisions of article L1121-8,
  - Adults made a ward of court, or who are unable to give their consent.

# EVALUATION CRITERIA

## Primary endpoint

The primary endpoint will be the rate of fall injuries following a loss of balance in each of the two groups (POTs *vs.* active control).

A fall injury will be recorded by the medic responsible for providing healthcare during the selection course. The injured participant will be required to attend an additional follow-up visit on the same day after the initial medical consultation.

## Secondary endpoints

Criteria for meeting the study’s four secondary objectives are as follows:

- - 1. The first secondary objective comprises two evaluation criteria: 1) self-reported scores on the Postural Awareness Scale (APPENDIX 3) pre- and post-intervention, and at the start of each session; 2) variation in measures of postural balance pre- and post-intervention.
    2. The second objective also comprises two criteria: 1) self-reported scores on Cohen’s Perceived Stress questionnaire (APPENDIX 3) pre- and post-intervention, and at the start of each session; 2) variation in measures of allostatic load in saliva pre- and post-intervention (the corticotropic axis: cortisol, DHEA, oxidative stress; the autonomic nervous system: alpha amylase and chromogranin A).
    3. The evaluation criterion for the third secondary objective will be the correct prediction of the risk of fall injury by the machine learning model.
    4. The evaluation criterion for the fourth secondary objective will be the rate of fall injuries in each of the two groups (POTs *vs.* active control), in the period between the end of the study and the end of the selection course. Information about when each participant left the course will be collected from the physician responsible.

# STUDY SCHEDULE

As the study unfolds, participants will be contacted at the following locations:

All measurements will be carried out on the premises of the respective military unit (the location of the selection course). Inclusion, and the end-of-study visit will be carried out at the respective military unit’s medical center. Both follow-up visits and POTs or active control sessions will be held at the same location as the selection course.

## Summary of patient follow-up

**Table 1 –** Summary of study visits.


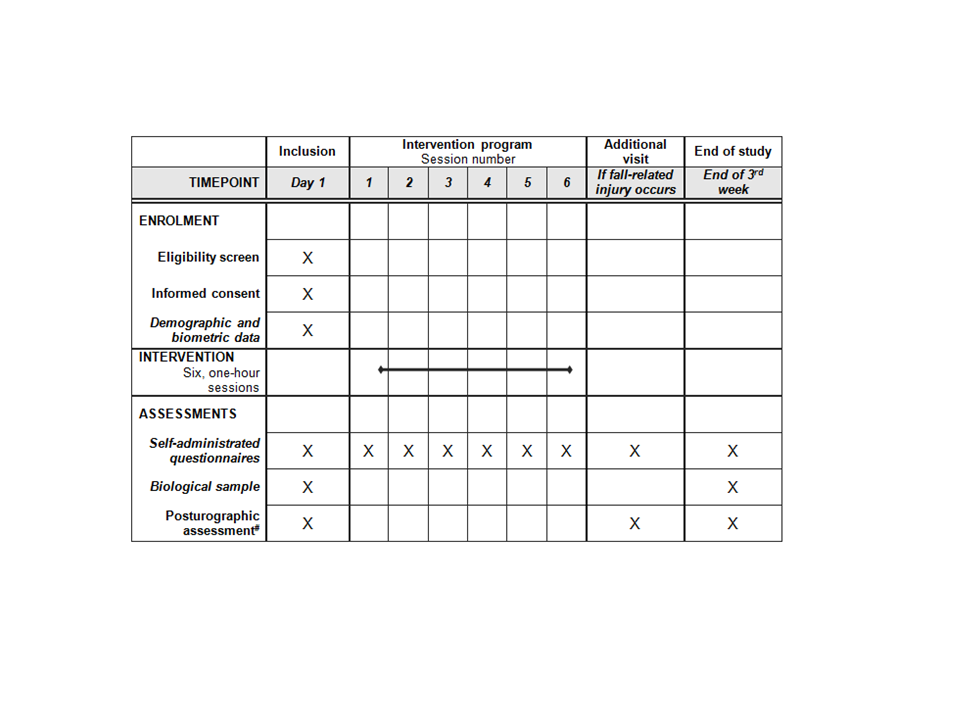


## Study visit schedule

The day before the start of the selection course, candidates will receive a group briefing from the investigator or one of his or her collaborators, together with a letter informing them about the study. Candidates will be given several hours to reflect on whether they would like to participate before the inclusion visit.

### Inclusion visit

The inclusion visit will take place the day after the group information session, and before any examinations related to the study. During the visit, the investigator or one of his or her collaborators will ask the participant if they want to take part in the study, and will inform him or her:

- of the objective of the study, and any constraints,
- foreseeable risks and expected benefits,
- that personal data collected during the study will be held and processed digitally, and state his/her rights regarding access, opposition and rectification,
- that samples will be conserved for scientific purposes after the study has ended, and obtain consent.

The investigator, or a colleague will answer any questions the participant may have. The latter will then have 15 minutes to reflect on his or her decision to take part in the study. Formal agreement will be obtained by signing the consent document (three copies).

Any changes to the study that impact either patient care, or benefits, risks and constraints will be presented in a new information document. The same procedure will be used to inform those concerned.

The various copies of the information document and the consent form will then be distributed as follows:

- A copy of the information document and the signed consent form will be given to the participant.
- The original will be held by the investigating medic in a safe place that cannot be accessed by third parties.

A copy of each consent form will be sent to the sponsor or its representative after inclusion or, at the latest, at the end of the study, in accordance with the procedures communicated.

### Initial evaluation visit

The initial evaluation visit will take place immediately after the inclusion visit for subjects who have given consent to participate. During this visit, the investigator, or one of his or her collaborators, will collect clinical data (age, height, weight and gender), run the BESS, and collect posturography measurements. The participant will then complete a medico-demographic questionnaire, together with questionnaires to measure postural awareness (the Postural Awareness Scale) and perceived stress (Cohen’s Perceived Stress questionnaire). Finally, a 4 ml saliva sample will be collected.

### Follow-up visits

A follow-up visit will be carried out just before each session of the POTs and active control interventions (the schedule for which is shown in Figure 4). During the follow-up visit, the participant will complete self-report questionnaires measuring postural awareness (the Postural Awareness Scale) and perceived stress (Cohen’s Perceived Stress).

Participants who suffer a fall trauma during the course will attend an additional follow-up visit, which will take place on the day of the injury. The military medic, or one of his or her assistants, will collect medical information relating to the trauma, and carry out a posturography measurement. The participant will then complete questionnaires measuring postural awareness (the Postural Awareness Scale) and perceived stress (Cohen’s Perceived Stress).

### End-of-study visit

Participant follow-up will end either: (*i*) on the day following the last session of the POTs or the active control intervention; or (*ii*) the day they leave the course (for whatever reason), if this occurs before the end of the intervention (POTs or active control).

A posturography measurement will be performed by the military medic, or one of his or her assistants, and the participant will complete questionnaires measuring postural awareness (the Postural Awareness Scale) and perceived stress (Cohen’s Perceived Stress). Finally, a 4 ml saliva sample will be collected.

## End of the study

### Discontinuation criteria for a participant

Participation will end:

- After the last session of the intervention (POTs or active control),
- On leaving the course (for whatever reason) if this occurs before the end of the intervention.

It will not be possible for a participant who leaves to re-join the program at a later date.

There are no exclusion criteria in the protocol.

### Early termination of the experimental procedure by the participant

All participants may withdraw from the study at any time, for any reason. In the event of early termination, data collected up to the date of withdrawal of consent may be used, unless the participant objects, in accordance with article L 1122-1-1 of the French Public Health Code. Participants who withdraw will not be replaced.

### Study discontinued by the sponsor

The sponsor may end the study at any time, for the following reasons:

- Inability of the investigator to include subjects on the planned schedule.
- No written consent.
- Major breaches of protocol.
- Incomplete or erroneous data.

The sponsor will draw up an end-of-trial declaration within 90 days of the end of the study.

If the clinical trial ends (definitively) earlier than planned, a declaration must be submitted to the ANSM and the Institutional Review Board within 15 days, giving the reasons for the decision.

### Study discontinued by the investigator

In the event of an adverse event that the investigator deems severe and likely to jeopardize the health of subjects, he or she may end the study in agreement with the sponsor.

## Research-related constraints and compensation for subjects

Participants may not simultaneously take part in another research project. No compensation will be given.

## Research schedule

- Duration of inclusion: 24 months
- Duration of the intervention: 3 weeks
- Duration of each participant’s participation: 4 weeks
- Total study time: 25 months

# LOGISTICS

## General logistics

The coordinating investigator, with the help of her scientific collaborator, will supervise all study visits (see paragraph 6.2). For inclusion and initial evaluation visits, the coordinating investigator and her scientific collaborator will be assisted by medical staff (physicians, nurses and stretcher-bearers) in the respective military unit. Follow-up visits will be carried out prior to each intervention session (POTs or active control) by the POTs instructor. The additional follow-up visit (carried out if the participant suffers a fall trauma during the study period) and the end-of-study visit will be conducted by the unit’s medical officer or one of his or her collaborators.

The coordinating investigator will also be responsible for monitoring progress, checking that progress reports are prepared, and that Case Report Forms are updated (requests for additional information, corrections, etc.).

## Experimental products or medical devices tested

No experimental products will be administered to participants, and no medical devices will be tested in the context of this study.

# VIGILANCE

## Definitions

An **adverse event** is defined as an unwanted occurrence in a person who participates in interventional research, whether or not it is related to the study, or the product to which the study relates.

An **adverse effect** is defined as an undesirable event for which a causal link (even weak) is suspected or can be established with a healthcare product or a medical procedure.

A **serious adverse effect (SAE)** is defined as any event or adverse effect that results in death, endangers the life of the person participating in the study, requires hospitalization or the prolongation of hospitalization, causes significant or lasting disability or incapacity, or results in a congenital anomaly or malformation, and, in the case of a drug, regardless of the dose administered.

A **serious incident** or **risk of incident** corresponds to the involvement of a medical device in an event that has resulted or may have resulted in the death or serious deterioration in the state of health of a patient, user or third party.

A **new fact** is any new data that may lead to a reassessment of the risk-benefit ratio of the study or of the product that is the object of the study, to changes in the use of this product, as the study is conducted, or in documents relating to the study, or to the suspension, interruption or modification of the research protocol or similar studies. In the case of trials involving the first administration to humans, or the use of a health product in people who have no known medical condition, any serious adverse effect is a new fact.

## Safety assessment methods and schedule

No specific parameters will be used to monitor patient safety. In accordance with standard care procedures, investigators must report adverse effects; incidents and the risk of serious incidents that they have identified or suspected throughout the patient’s participation in the study.

## Procedures for reporting adverse effects, incidents or the risk of incidents

As soon as an investigator becomes aware of an adverse effect, an incident or the risk of a serious incident, he or she must report it directly to the relevant authority (see Table below) in accordance with the procedures in force at the establishment, specifying in the report that the patient is included in the POSITION study.

| Event related to: | Health monitoring or agency concerned | Relevant authority |
| --- | --- | --- |
| A drug | Drug safety | Regional Drug Safety Center (Centre Régional de Pharmacovigilance) |
| A blood product | Hemovigilance | Local hemovigilance center (Correspondant local d’hémovigilance) |
| A medical device | Equipment safety | Local equipment safety center |
| A care-related act | No health surveillance.  Direct transmission to → | Regional Health Agency (Agence Régionale de Santé) * |
| A care-related infection | Infection control | Local infection control center |
| A non-drug toxic substance | Toxicovigilance | Poison Control Center (Centre Antipoison) |
| A psychogenic substance leading to addictive behavior or drug dependence | Addiction surveillance | Drug Dependence Assessment Center (Center d’Evaluation et d’Information sur les pharmacodépendances-addictovigilance) |
| Food, a food supplement, adulterated products | Toxicovigilance | Poison Control Center (Centre Antipoison) |

* In the case of adverse effects related to healthcare, including venous samples used in the study, they will only be transmitted to the Regional Health Authority if they meet the criteria set out in the Order of February 20, 2017 relating to the criteria for reporting to the Regional Health Authority alerts collected by members of the regional vigilance and support network [(](https://www.legifrance.gouv.fr/eli/arrete/2017/2/20/AFSP1705441A/jo/texte)https://www.legifrance.gouv.fr/eli/arrete/2017/2/20/AFSP1705441A/jo/texte).

Any report of an adverse effect must state that the patient is participating in the study, and must be recorded in the Case Report Form.

In all cases, the report may be transmitted either via the establishment’s internal reporting system (if applicable), or via the adverse event reporting portal.

https://signalement.social-sante.gouv.fr/psig_ihm_utilisateurs/index.html#/accueil.

This portal forwards the report to the relevant authority, and sends the notifier an acknowledgement of receipt, which can be included in the patient’s file.

In the event of a new fact, the investigator, the person responsible for vigilance, and the sponsor will discuss any safety measures that may need to be implemented, the continuation of the study, and the need to contact the Regional Health Authority.

## Methods and duration of follow-up of participants following an adverse effect

When an adverse effect persists, or an incident has ongoing consequences, including after the study has ended, the investigator will monitor the person participating in the study until the event is considered resolved, and will report any follow-up data to the relevant authority.

## Late emergence of serious adverse effects

If the investigator becomes aware of an adverse effect that occurs after the patient’s follow-up period has ended, he/she will report it immediately to the relevant authority, using the same procedures.

# BIOLOGICAL SAMPLE COLLECTION

Within the framework of the study, we will create a biological collection to meet the stated research objectives, and to be able to carry out other analyses or assays in the light of new facts brought to light by the literature, while the purpose of the study will remain unchanged. Analyses carried out for research purposes will not be used for diagnostic purposes.

The saliva samples that are collected (2 x 2 ml bottles) during inclusion and end-of-study visits will be stored on site at −20°C and then transported under optimal temperature conditions by the investigating team to be stored in the −80°C freezers at IRBA (in a dedicated room, 1 place du Général Valérie André, 91223 Brétigny-sur-Orge) until they are analyzed.

This will result in a collection of 1,600 samples (2 bottles, 2 measurement times, 400 subjects).

The study’s principal investigator is responsible for saliva sampling.

Each sample will be identified by a code (affixed to cryogenic labels) as follows:

- Subject code: Unit code + Subject number

- Visit number: inclusion visit: Vin; for follow-up visits: V1, V2, V3 and V4; final visit: Vfin. Additional visits will be identified by the code Vadd1, Vadd2, etc., depending on the number of additional visits carried out.

The collection of biological samples undertaken in the context of this study has been declared to the competent authority. After the study has ended, the conservation of the collection of biological samples will be declared to the minister in charge of research and to the director of the Agence Régionale d’Hospitalisation (and submitted to the Institutional Review Board for an opinion if the purpose of the study changes).

# DATA COLLECTION AND PROCESSING

Any original document or object that can be used to prove the existence or accuracy of data or facts recorded during the study is defined as a source document.

Source documents include Case Report Forms, paper-based questionnaires, and digitized clinical and paraclinical data.

## Data collected

### Clinical parameters

Clinical data collection will be limited to injury history, age, sex, height and weight. These data will be recorded using posturography software.

### Para-clinical parameters

Postural balance will be measured by a clinical test (the BESS) and posturography.

1. The Balance Error Scoring System (BESS)

The BESS assesses static postural balance (eyes closed) in six conditions, using three positions (double leg stance *vs.* single leg stance *vs.* tandem stance) on two surfaces (firm *vs.* soft) (Figure 5). The participant must remain balanced without moving for 20 seconds. Points are given for postural errors (e.g., eye opening, hip flexion greater than 30°, etc.). The sum of the points obtained in the six conditions gives the total BESS score (Docherty et al., 2006)

 
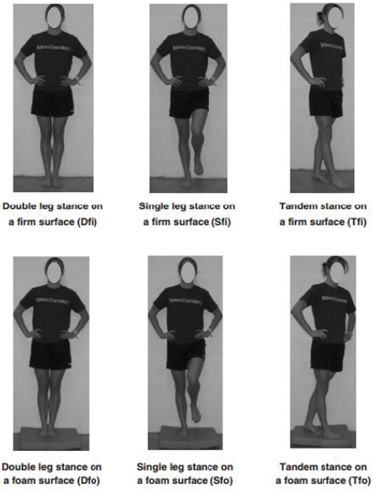


**Figure 5 –** The Balance Error Scoring System is a clinical test that assesses static postural balance in six conditions, using three positions (double leg stance *vs.* single leg stance *vs.* tandem stance) on two surfaces (firm *vs.* soft).

1. Posturography

Posturography is the recording of static postural balance using a stabilometric platform (FEETEST 6^©^, Techno Concept^®^, France) (Figure 6). It consists of four small platforms that locate the position of the body’s center of pressure from the coordinates of four points of contact (left heel, left metatarsal, right heel and right metatarsal). Postural recording takes place with eyes closed, and lasts approximately one.


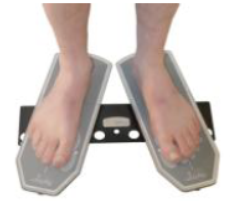


**Figure 6** – The FEETEST 6^©^ platform (Techno Concept^®^).

To ensure reproducibility in measurements of para-clinical parameters, investigators from the units involved in the initial assessment visit will be trained in how to carry out the BESS test (using a video). Similarly, a standard procedure will be put in place for posturography measurements.

1. Psychometric data

Self-report questionnaires will be used to measure postural awareness (the Postural Awareness Scale) and perceived stress (Cohen’s Perceived Stress Scale).

- 1. The Postural Awareness Scale (Cramer, Mehling, Saha, Dobos, & Lauche, 2018)

This questionnaire evaluates body awareness involved in postural control. A French version is currently being validated. The questionnaire is made up of 12 items, each rated from 1 (not at all true about me) to 7 (very true about me). The questionnaire assesses two dimensions of postural awareness: (*i*) Ease/Familiarity with Postural Awareness; and (*ii*) Need for Attention Regulation with Postural Awareness.

- 1. Cohen’s Perceived Stress Scale (Cohen, Kamarck, & Mermelstein, 1983)

This questionnaire evaluates how individuals rate their perception of stress. It consists of 14 items, each rated from 1 (never) to 5 (often). A high score on the questionnaire indicates a high level of perceived stress.

The two questionnaires are presented in APPENDIX 3. Participants will complete paper-based versions.

### Biological parameters

A saliva sample will be collected during the initial evaluation visit, and the end-of-study visit (2 x 2 ml at each visit). These samples will be used to monitor markers of the corticotropic axis (cortisol and DHEA), and the autonomic nervous system (alpha-amylase and chromogranin).

## Data collection methods

A paper-based Case Report Form will be created for the study.

The person(s) responsible for maintaining Case Report Forms must be clearly identified in the task allocation document. Each Case Report Form will be strictly anonymous and labelled using the participant’s identification number. During the study, or when it ends, the data collected and transmitted to the sponsor by the persons directing and supervising the research (or any other specialist) will be anonymized. Under no circumstances can the names or addresses of the persons concerned appear in clear text.

## Data flow

Clinical data will be recorded in a database, consistent with the data recorded in the paper-based Case Report Form.

All data recorded in the Case Report Form and *via* questionnaires will be entered by optical reading of paper-based documents (OMR Manager software), and inconsistent data will be checked manually. These data, together with data from various salivary assays, will be consolidated in an anonymized database held on a secure computer with up-to-date security software. Regular back-ups will be made on secure removable media. This medium will be regularly checked for viruses.

Access to this data will be restricted to authorized persons (investigators and collaborators).

The physical security of all materials will be ensured by keeping them in locked premises on a guarded military compound.

## Data access and confidentiality

The sponsor is responsible for obtaining the agreement of all parties involved in the study in order to guarantee direct access to all study locations, source data, source documents and reports needed for quality control and audit purposes.

The persons directing and supervising the study will make any documents and individual data that are essential for monitoring, quality control and auditing the study available to persons authorized to access these documents in accordance with the legislative and regulatory provisions in force.

In accordance with current legislation, in particular Articles L.1121-3 and R.5121-13 of the French Public Health Code, persons with direct access to source data (for example, investigators, quality control personnel, instructors, clinical research assistants, auditors and any other persons called upon to collaborate in the trials) will take all necessary precautions to ensure the confidentiality of information relating to the study, and those who participate, notably their identity, and the results obtained. These persons, together with those directing and supervising the study, are bound by the conditions of physician–patient privilege.

During, or at the end of the study, individual data that is collected and reported to the sponsor by the persons directing and supervising the study (or any other specialized parties) will be codified. Under no circumstances should the names or addresses of the persons concerned appear in plain text.

Participants will be coded as follows: unit code (GIGN – GI, CPA 10 – CP, 1^ier^ RPIMA –RP, 13^ième^ RDP –RD, ECOFUS – EC, BSPP – BS); a number indicating the order of inclusion in the unit; the first letter of the participant’s surname and first name.

The sponsor will ensure that each person involved in the study has been informed about access to personal data, which is essential for quality control purposes.

# STATISTICAL ASPECTS

## Calculating the number of subjects required

To date, there are no data in the literature on the effectiveness of POTs in preventing sports-related injuries. However, discussions with military unit managers and medical officers suggest that a reduction in the injury rate of around 15% would be clinically relevant. Preliminary epidemiological data from the various units indicates that the rate of fall injuries is around 50%.

To determine the sample size required to observe a significant difference in rates between the two groups, we used the following parameters:

- statistical test: two-tailed
- fall rate in the active control group: 50%
- fall rate in the POTs group: 35%
- statistical significance = 0.05
- power = 0.80

Taking into account potential drop-outs (estimated at 10%), the sample should consist of 200 participants per group, for a total of 400 participants over the two years of the study.

## Statistical methods

Statistical analyses will be carried out using MATLAB 2020b, R version 3.5.2, and Statistica software.

The risk of error will be set at 5%, and two-tailed tests will be used.

Missing data will be replaced by the mean of the variable in the sample for the group under consideration (POTs or active control).

First, the number of participants will be described in a flow chart (included and excluded subjects, reasons for exclusion, subjects included, or not, in the analysis and reasons for exclusion). A descriptive analysis will then be run on all of the variables collected, and for each of the two groups (POTs *vs.* active control). Qualitative variables will be described using frequencies and percentages, while quantitative variables will be described using mean and standard deviation, or medians and interquartile ranges.

**Analysis of the main objective**

The effect of the intervention (POTs *vs.* active control) on the rate of fall injuries will be evaluated using a comparative survival analysis (the Cox model, with clinical variables as potential confounders). Survival curves for POTs and active control groups will be compared using the log-rank test.

**Analysis of secondary objectives**

For the first secondary objective, the effect of the POTs intervention on postural awareness will be evaluated by comparing the following for the POTs group: (*i*) scores on the Postural Awareness Scale at the six measurement times (repeated measures ANOVA), and (*ii*) posturography measurements pre- and post-intervention (paired samples comparison of means).

For the second secondary objective, the effect of the POTs intervention on the psychobiological stress response will be evaluated by comparing the following measures for the POTs group at all six measurement times: (*i*) scores on Cohen’s Perceived Stress Questionnaire (repeated measures ANOVA), and (*ii*) biological stress variables (paired samples comparison of means).

For the third secondary objective, machine learning will be used to evaluate the accuracy of the predictive model of the risk of fall injury using the following calculation:

|  | $\text{Accuracy=}\frac{\text{True }\text{Fall-related injury }\text{participants + True }\text{Non-fall }\text{participants}}{\text{Total number of participants}}$ |  |
| --- | --- | --- |

where N_prediction of trauma_ is the number of participants for whom the machine learning model predicts the occurrence of a fall injury and who do, in fact, suffer a fall injury during the selection course; N_prediction of_ _good health_ is the number of participants for whom the model predicts the non-occurrence of a fall injury and who do not, in fact, suffer a fall injury during the selection course; and N_total_ is the total number of participants included in the study.

For the fourth secondary objective, the long-term effectiveness of the POTs intervention in preventing fall injuries between the end of the study and the end of the selection course will be evaluated using a comparative survival analysis (the Cox model). Survival curves for POTs and active control groups will be compared using the log-rank test.

# QUALITY CONTROL AND ASSURANCE

## Quality control

A clinical research associate will be appointed by the sponsor. This person will visit each facility on a regular basis: when the study is set up, once or several times during the course of the study, depending on the rate of inclusion, and at the end of the study. The elements to be reviewed during these visits, and their frequency, will be defined before the study is set up, in collaboration with the coordinating/principal investigator and the scientific manager, and in accordance with the study’s risk assessment.

The study has been classified as an interventional study with minimal risks and constraints, falling under risk category B.

During these visits, the following elements will be reviewed:

- compliance with the research protocol, the procedures defined therein and the regulatory texts in force,
- the quality of data recorded in the Case Report Form: accuracy, missing data, consistency of data with source documents (medical records, appointment books, original laboratory results, etc.),
- the management of any products and samples that may be collected.

A written report will be drawn up following each visit.

## Audit and inspection

An audit may be carried out at any time by persons mandated by the sponsor, and independent of those in charge of the study, or during an inspection conducted by health authorities or the French Data Protection Authority (Commission Nationale Informatique et Libertés). The aim is to ensure the quality of the study, the validity of its results, and compliance with any legislation and regulations in force.

Auditors/inspectors must be given direct access to source and medical data and any other documents relevant to the conduct of the clinical study.

Data confidentiality and participant anonymity will be respected.

The persons who direct and supervise the study agree to comply with the requirements of the sponsor and the competent authority with regard to an audit or inspection.

The audit can be applied to all stages of the study, from the development of the protocol to the publication of the results, and the classification of data used or produced in the context of the study.

## Commitment to responsibility

Before the study begins, each investigator will provide an up-to-date, dated and signed curriculum vitae (CV) to the sponsor’s representative. The CV will include an RPPS number (The Directory of Health, Social work and Medico-Social Professionals in France), the number the person is registered under with the Ordre des Médecins (the French National Medical Council) (excluding military physicians), and details of any previous participation in studies and training related to clinical research.

Each investigator agrees to comply with legislative and regulatory obligations, and to conduct the study in accordance with regulations, and in compliance with the current Declaration of Helsinki. The principal investigator will sign an undertaking of responsibility, which will be given to the sponsor’s representative.

The investigators and their collaborators will sign a form that designates the allocation of tasks and specifies their role, and each person will provide a copy of their CV.

# ETHICAL AND REGULATORY CONSIDERATIONS

## Ethical justification

### The distinction between care and research

In the context of the POSITION project, both POTs and active control interventions, together with all measurements carried out, relate to research activities.

### Benefit/ risk ratio

#### Benefits

Individual benefit(s):

The POSITION project will test the effectiveness of a POTs intervention in preventing fall injuries during physical activities in the military context. In addition, the study will seek to describe the risk and protective factors that moderate the occurrence of an injury, including cognitive mechanisms involved in postural awareness, and the psychobiological stress response. The outcomes of this study are expected to support the development of new, evidence-based prevention/medical strategies that can predict the risk of fall-related injury during physical activities in the military context, together with their prevention due to a POTs intervention. The expected benefits of this study relate to the protection of the health of combatants in the context of their military service.

Group benefit(s):

On a group level, the prevention of fall injuries will improve the health of the military population, and consequently their ability to carry out their missions. Benefits relate to both the health and the safety of combatants in the context of their military service.

#### Risks

Individual risk(s)

No individual risk directly related to the POSITION protocol can be identified.

Group risk(s)

No group risk directly related to the POSITION protocol can be identified.

#### Benefit/risk ratio

The benefit/risk ratio is positive, as the study has both individual and group benefits, and there no risks can be identified that are directly related to the POSITION protocol.

### Methodological choices

In the context of the POSITION project, all measurements are related to the research being carried out; therefore, no additional monitoring modalities are planned.

## Compliance with regulations and legislation

The study will be conducted in accordance with the present protocol, and in compliance with the Declaration of Helsinki (as modified in Fortaleza in 2013 Cf. full version), Good Clinical Practice recommendations (GCP, ICHE6) and any locally applicable regulations.

As this study falls within the definition given in 2° of Article L.1121-1 of the French Public Health Code, it requires approval from an Institutional Review Board.

In accordance with the conditions set out in Article L 1121-10 of the French Public Health Code, the French State assumes responsibility for compensating those involved for any harmful consequences of the study.

In the event of a favorable decision, the sponsor will forward the opinion of the Institutional Review Board and the summary of the protocol to the French Medicines Agency (Agence nationale de sécurité du médicament et des produits de santé) for information.

The data recorded during this study will be digitally processed at IRBA in compliance with Law No. 78-17 of January 6, 1978 relating to information technology, files and freedoms as amended by Law 2018-493 of June 20, 2018 and the General Data Protection Regulation (Articles 6.1.f, 9. *j* and Article 13).

This study falls within the scope of the ‘Reference Methodology’ (MR-001) following the application of the provisions of Article 73 of the amended law of January 6, 1978 relating to information, files and freedoms. The French Defence Health Service Headquarters (Direction centrale du service de santé des armées), the sponsor of this study, has signed a commitment to comply with this Reference Methodology.

The data controller for the French Armed Forces Medical Corps is the Central Director of the Armed Services Health Service. The Data Protection Officer (DPO) is the Director of Legal Affairs of the Ministry of the Armed Forces. The data controller’s representative, who is the contact point for the ministerial DPO, can be contacted at ssa.rrt.fct@intradef.gouv.fr.

## Protocol amendment

Any substantial modification, in other words, any modification that is likely to have a significant impact on the protection of individuals, the validity and outcomes of the study, the interpretation of supporting scientific documents, or the methods used, must be the subject of a written amendment submitted to the sponsor, who must obtain a favorable opinion from the Institutional Review Board prior to its implementation.

Non-substantial modifications, in other words, those that do not have a significant impact on any aspect of the study, must be communicated to the Institutional Review Board for information purposes.

All amendments to the protocol must be brought to the attention of all healthcare professionals taking part in the study, who undertake to respect them.

# CONSERVATION OF DOCUMENTATION AND DATA

The following documents relating to this study will be archived in accordance with Good Clinical Practice **for a period of 15 years** following the end of the study:

- By the investigators:
- The protocol and any amendments to the protocol,
- A copy of participants signed informed consent forms,
- Case Report Forms,
- Participants’ personal files,
- All other research-related documents and correspondence.

All these documents are the responsibility of the physician for the duration of the regulatory archive period.

- By the sponsor:
- The protocol and any amendments to the protocol,
- A copy of participants signed informed consent forms,
- Original Case Report Forms,
- All other research-related documents and correspondence.

All of these documents are the responsibility of the sponsor for the duration of the regulatory archive period.

Nothing may be moved or destroyed without the sponsor’s consent. At the end of the regulatory archive period, the sponsor will be consulted before destruction, and must give written agreement. All data, documents and reports may be subject to audit or inspection.

# PUBLICATION RULES

## Scientific communications

Data analysis will be carried out by the IRBA. This analysis will result in a written report that will be submitted to the French Defense Health Service Headquarters (Direction centrale du Service de santé des armées). This report will be used in the preparation of one or more publications.

Any written or oral communication of the outcomes of this study must receive prior approval from the person who directs and supervises it, and, where applicable, any committee set up in the context of the project.

The report of the main results will include the name of the sponsor (Direction centrale du Service de santé des armées), all the healthcare professionals who included or monitored patients, the methodologists, biostatisticians and data managers who took part, and members of related committee(s). International reporting and publication rules will be followed (Vancouver Convention, February 2006; Recommendations for the Conduct, Reporting, Editing, and Publication of Scholarly Work in Medical Journals Updated, IJCME, 2018).

## Communicating results to patients

In accordance with Article L1122-1 of the French Public Health Code, patients will be informed of the overall results of the study during an oral presentation in the various military units.

## Data handover

Data collection and management is the responsibility of the IRBA. Conditions for the handover of all or part of the study’s database will be decided by the Direction centrale du Service de santé des armées (the sponsor) and will be the subject of a written contract.

1. REFERENCES

Amboni, M., Barone, P., & Hausdorff, J. M. (2013). Cognitive contributions to gait and falls: evidence and implications. *Movement disorders, 28*(11), 1520-1533.

At, W. (2016). *Epidémiologie de la traumatologie aigue sportive chez les sapeurs-pompiers en phase d'incorporation.* (Thèse de médecine générale), Université Paris 13, Paris.

Bahr, R., & Holme, I. (2003). Risk factors for sports injuries--a methodological approach. *British Journal of Sports Medicine, 37*(5), 384-392. doi:10.1136/bjsm.37.5.384

Bauvent, Y. (2014). *Etude de la tolérance et de l’état de fatigue au cours de la préparation physique initiale des EVAT au CFIM de Gap. Etude de l’effet sur les capacités en endurance et en force.* (Thèse de médecine générale), Université Aix Marseille, Marseille.

Bertrand, M. (2016). *Pathologies traumatiques au cours du stage commando marine : analyse des interactions entre vigilance et proprioception.* (Thèse de médecine générale), Université Paris XI, Paris.

Bigard, X., Cravic, J. Y., & Banzet, S. (2010). Prévention des risques liés à la préparation physique du militaire: synthèse des connaissances actuelles. *Médecine et Armées, 38*(1), 07-16.

Brocard, M. (2014). *Pathologies traumatiques au bataillon des Marins Pompiers de Marseille, étude prospective de juin 2012 à avril 2013.* (Thèse de médecine générale), Université Aix Marseille, Marseille.

Chipault, M. (2016). *Epidémiologie de la pratique sportive et des blessures liées aux activités physiques et sportives chez les personnels naviguants militaires français.* (Thèse de médecine générale), Université Paris-Sud, Paris.

Cohen, S., Kamarck, T., & Mermelstein, R. (1983). A global measure of perceived stress. *Journal of health and social behavior*, 385-396.

Craig, A. D. (2002). How do you feel? Interoception: the sense of the physiological condition of the body. . *Neuroscience, 3*(8), 655-666.

Cramer, H., Mehling, W. E., Saha, F. J., Dobos, G., & Lauche, R. (2018). Postural awareness and its relation to pain: validation of an innovative instrument measuring awareness of body posture in patients with chronic pain. *BMC musculoskeletal disorders, 19*(1), 109.

Crosnier, S. N. (2013). *Évaluation du sommeil des sous-mariniers en situation opérationnelle sur Sous-marins Nucléaires Lanceurs d’Engins : Influence des Techniques d’Optimisation du Potentiel sur le sommeil. .* (Doctorat en médecine),

Delahunt, E., Bleakley, C. M., Bossard, D. S., Caulfield, B. M., Docherty, C. L., Doherty, C., . . . Gribble, P. A. (2018). Clinical assessment of acute lateral ankle sprain injuries (ROAST): 2019 consensus statement and recommendations of the International Ankle Consortium. *British Journal of Sports Medicine, 52*(20), 1304-1310. doi:10.1136/bjsports-2017-098885

Dirand, E. (2014). *Préparation mentale : réinstaurons l’indispensable duo « santé-performances » de nos soldats*. (Mémoire professionnel de M2 STAPS, spécialité EMIS).

Docherty, C. L., Valovich McLeod, T. C., & Shultz, S. J. (2006). Postural control deficits in participants with functional ankle instability as measured by the balance error scoring system. *Clin J Sport Med, 16*(3), 203-208.

Etat-major des armées - Centre national des sports de la défense. (2011). *Manuel d’Entrainement Physique Militaire et Sportif*.

Forbes, P. A., Chen, A., & Blouin, J. S. (2018). Sensorimotor control of standing balance. In *Handbook of Clinical Neurology* (Vol. 159, pp. 61-83): Elsevier.

Gage, W. H., Winter, D. A., Frank, J. S., & Adkin, A. L. (2004). Kinematic and kinetic validity of the inverted pendulum model in quiet standing. *Gait & Posture, 19*(2), 124-132.

Longin, A. (2015). *Mécanismes de survenue des traumatismes des membres inférieurs au cours du stage commando marine.* (Thèse de médecine générale), Université Brest - Bretagne Occidentale, Brest.

Meeuwisse, W. H. (1994). Assessing causation in sport injury: a multifactorial model. *Clin J Sport Med, 4*, 166–170.

Mehling, W. E., Gopisetty, V., Daubenmier, J. J., Price, C. J., Hecht, F. M., & Stewart, A. (2009). Body awareness: construct and self-report measures. *PLoS One, 4*(5), e5614.

Millet, C. (2013). *Influence des Techniques d’Optimisation du Potentiel sur la préparation mentale à l’éjection chez des pilotes.* Mémoire de diplôme universitaire.

Morinière, N. (2013). *Etude prospective des pathologies médicales et traumatiques au cours du stage commando marine et proposition de mesures de prévention.* (Thèse de médecine générale), Université Brest-Bretagne occidentale, Brest.

Pagninia, F., Manzonib, G. M., Castelnuovoab, G., & Molinariab, E. B. (2013). A brief literature review about relaxation therapy and anxiety. *Body, Movement and Dance in Psychotherapy, 8*(2), 71-81.

Pleche, S. (2018). *Étude des pathologies sportives en lien avec la formation militaire initiale chez des jeunes engagés du Centre de Formation des Militaires du rang d’Angoulême, et engagés sous-officiers de l’École de Saint-Maixent.* (Thèse pour le doctorat en médecine), Université de Bordeaux, Bordeaux.

Ressort, T., Desjeux, G., Marsan, P., & Thevenin-Garron, V. (2013). Les affections en service liées aux sports chez les militaires français. *Santé publique, 25*(3), 263-270.

Steiler D., D. J., Trousselard M. . (2011). *Developing positive emotions for the improvement of first year students’ well-being*. Paper presented at the International Congress of Positive Psychology, Philadelphie, USA.

Trousselard, M., Fidier, N., Ferhani, O., & Perraut-Pierre, E. (2010). *Détermination d’un outil de mesure de l’impact des stress vécus en fonction de la résilience des individus : intérêt pour l’évaluation de l’efficacité des mesures de prévention.*, (Rapport de synthèse intermédiaire du contrat d’objectif 10co708, I. Brétigny-sur-Orge).

Trousselard M., P. E., Ferres Saint Aubin K. (2010). *Impact of positive emotions enhacement on physiological processes and psychological functioning in military pilots.* . Paper presented at the Human factor & medicine panel symposium (HFM), , Sofia, Bulgaria.

Vealey, R. S., & Greenleaf, C. A. I. (2010). Seeing in believing: understanding and using imagery in sport. In J. M. Williams (Ed.), *Applied sport psychology: personal growth to peak performance* (pp. 267-304).

Verdonk, C., Duffaud, A., Longin, A., Bertrand, M., Canini, F., & Trousselard, M. (2019). *Equilibre postural et risque de blessure au stage commando marine*. Paper presented at the XIVème Journée des internes et des assistants, Ecole du Val-de-Grâce (Paris).

Verdonk, C., Trousselard, M., Medani, T., Vialatte, F., & Dreyfus, G. (2020). Probing the posture with machine learning supports the enhanced body awareness hypothesis in trait mindfulness. *(submitted)*.

Volchan, E., Rocha-Rego, V., Bastos, A. F., Oliveira, J. M., Franklin, C., Gleiser, S., . . . Figueira, I. (2017). Immobility reactions under threat: A contribution to human defensive cascade and PTSD. *Neurosci Biobehav Rev, 76*(Pt A), 29-38. doi:10.1016/j.neubiorev.2017.01.025

William, J. M., Zinsser, N., & Bunker, L. (2010). Cognitive techniques for building confidence and enhancing performance. In J. M. William (Ed.), *Applied sport psychology: personal growth to peak performance* (pp. 306-355).

Williams, J. G. P. (1971). A etiological classification of injuries in sportsmen. *British Journal of Sports Medicine, 5*(4), 228-230.

World Health Organization. (2003). *Health and development through physical activity and sport*. Retrieved from

1. Appendixes

Appendix 1 : Summary of studies evaluating the effects of POT.

| **Population** | **Performance / Target** | **Results / Limitations** | **Reference** (if available) | **POT Program** |
| --- | --- | --- | --- | --- |
| Students in 1st year of medicine (officer cadet) and business school | Cognitive performances | Benefits of POT. on feeling of efficiency in preparation for a competitive examination/exam  Limitations: absence of control | (Steiler D., 2011) | 8 weeks  1h/week + daily practice exercises |
| Air force pilots | Cognitive performance | Benefit of T.O.P. on ejection decision making in simulator  Limit: 5 subjects | (Millet, 2013) | Memory not searchable |
| 16th BC on Sentinel mission | Psycho-cognitive and physical performance, stress level | Project PHYTOP  Benefits on cognitive performance (memory test) and improvement in stress tolerance resources (optimism and motivation) | Non publié | Unpublished Training 1h30 weekly combined with 1h30 supervised sports practice - 9 weeks |
| Paris Fire Brigade | Stress management | Effectiveness of techniques on perceived stress, negative mood, local immunity and post-traumatic stress measurement.  Cardiac Coherence (CC) seems to be more effective on immunity, while POT is more effective in reducing perceived stress.  Single-center randomized study | (Trousselard, Fidier, Ferhani, & Perraut-Pierre, 2010) | Regular training for 2 months |
| Submariners in operational situations on nuclear-powered submarines | Stress management / Sleep management  Association POT and CC | Benefits of POT for both on-watch and off-watch submariners.  Mixed evaluation: subjective for mood disorders and objective for sleep measurement in operational conditions | (Crosnier, 2013) | Dissertation not available |
| Air Force pilots on OPEX | Operational performance and perceived stress on mission | Afghanistan Decrease in perceived stress, feelings of fatigue, urinary cortisol excretion, as well as improved group cohesion, particularly in squadron leader cohesion (vertical cohesion) for the two squadrons that benefited from these techniques.  Randomized study | (Trousselard M., 2010) | 8 weeks  1h/week + practice exercises between 2 sessions |
| Infantry soldiers | Operational performance | Benefits of short, intense TOP training (two hours daily) on motor performance (rope climbing) and technical performance (FAMAS assembly and disassembly) | (Dirand, 2014) | 2 hours daily - over a short period |

Appendix 2: Details of the programs’ contents

**Sessions - Active Control Group**

| **Session number** | **Main idea** | **Cognitive process** | **Activity/ activities** | **Organization of the session**  **(duration, group)** |
| --- | --- | --- | --- | --- |
| 1 | Presentation and identification of the different sensory channels | Perception and attention | Presentation of VAKOG  (Visual, Auditory, Olfactory and Gustatory)  Identification of the main sensory channel  Example applications | 45–60 minutes  In groups of 20/25 people maximum  Practical exercises in groups of 5 to 10 participants, depending on the activity |
|  | Integration of information | Memory /  Working memory | Presentation of memory processes, with a focus on working memory  Example of an optimization tool: the Memory Palace  Practical exercises working in pairs and groups |  |
| 3 | Reasoning 1 | Reasoning, coping strategy | Presentation of the different types of reasoning  Individual and group ‘psycho-technical’-type exercises |  |
| 4 | Reasoning 2 | Conceptualization, expression and communication of ideas | Dissertation topics: immortality, new technologies in the military, self-sacrifice, emotions and taking action, etc. |  |
| 5 | Mental procedure before taking action | Mental imaging | Presentation of mental imagery, its use in the professional context and performance objectives (example of the PAF and high-level athletes)  Individual exercises (the tower of Hanoi), then as a group (the marshmallow challenge) |  |
| 6 | Faced with a problem, using imagination and creativity as a solution | Imagination, divergent and convergent thinking | Presentation of the theme ‘creativity and active thinking’  Individual problem-solving exercises and inter-group challenges |  |

* Divergent thinking: producing many ideas from one stimulus. Convergent thinking: producing a single solution that integrates several elements.

**Sessions - ORAF Group**

| **Session number** | **Theme** | **Techniques worked on** | **Content** | **Organization of the session**  **(duration, group)** |
| --- | --- | --- | --- | --- |
| 1 | ORAF Presentation - Breathing | Breathing - Relaxation | Presentation of the ORAF: philosophy, theory & application  Breathing: theory and practice | 45 to 60 minutes  In groups of up to 20 people |
| 2 | Sleep and Vigilance | Relaxing breathing and calm imagery | Sleep/Vigilance/Fatigue theory  Continual awareness of the whole body through relaxation (recovery and relaxation); using VAKOG to support an internal dialogue/relaxing imagery |  |
| 3 | Stress | The optimized activation signal (OAS) technique | Theoretical presentation of stress  Implementation of the OAS technique |  |
| 4 | Self-confidence, performance optimization | Relaxing breathing  Mental rehearsal | Mental rehearsal: presentation and practical exercise |  |
| 5 | Mindfulness and performance optimization | Geolocation  (taking the environment into account)  Dynamic breathing | Practical geolocation exercise, optimized complete dynamization and immediate mental projection |  |
| 6 | Motivation and performance | Relaxation  Mental projection of goals (MPG) | Motivation theory + MPG  Individual relaxation exercise, followed by an MPG exercise |  |

Appendix 3 : PAS and PSS questionnaires

| **Questionnaire PAS** | | 1  Ne me correspond pas du tout | 2 | 3 | 4  Me correspond moyennement | 5 | 6 | 7  Me correspond fortement |
| --- | --- | --- | --- | --- | --- | --- | --- | --- |
| 1 | J'ai besoin d'être très concentré(e) pour prendre conscience de ma posture corporelle |  |  |  |  |  |  |  |
| 2 | Quand je me tiens dans une mauvaise posture corporelle, souvent je ne la remarque pas avant qu'elle devienne douloureuse |  |  |  |  |  |  |  |
| 3 | Quand je suis assis(e), j’ai souvent tendance à m’avachir |  |  |  |  |  |  |  |
| 4 | Quand je me concentre sur une activité spécifique, je prends souvent une posture corporelle particulière sans m'en rendre compte |  |  |  |  |  |  |  |
| 5 | Quand je me concentre sur une activité spécifique, je prends souvent une posture corporelle particulière sans m'en rendre compte |  |  |  |  |  |  |  |
| 6 | Quand je me concentre sur une activité spécifique, je prends souvent une posture corporelle particulière sans m'en rendre compte |  |  |  |  |  |  |  |
| 7 | Au travers de ma posture corporelle, je peux intentionnellement modifier l'impression que je donne aux autres |  |  |  |  |  |  |  |
| 8 | Tout au long de la journée, je suis en permanence conscient(e) de la façon dont je suis assis(e) ou debout |  |  |  |  |  |  |  |
| 9 | Je suis souvent conscient(e) / me rends compte de ma posture corporelle du moment, que je sois assis(e) ou debout |  |  |  |  |  |  |  |
| 10 | Même si je suis focalisé(e) sur quelque chose, je suis en permanence conscient(e) de ma posture corporelle |  |  |  |  |  |  |  |
| 11 | Au travers de ma posture corporelle, je peux contrôler consciemment mon humeur |  |  |  |  |  |  |  |
| 12 | Je remarque si ma posture corporelle est bonne pour moi, ou non, seulement quand je me concentre dessus |  |  |  |  |  |  |  |

**Questionnaire de Stress perçu de Cohen**

**
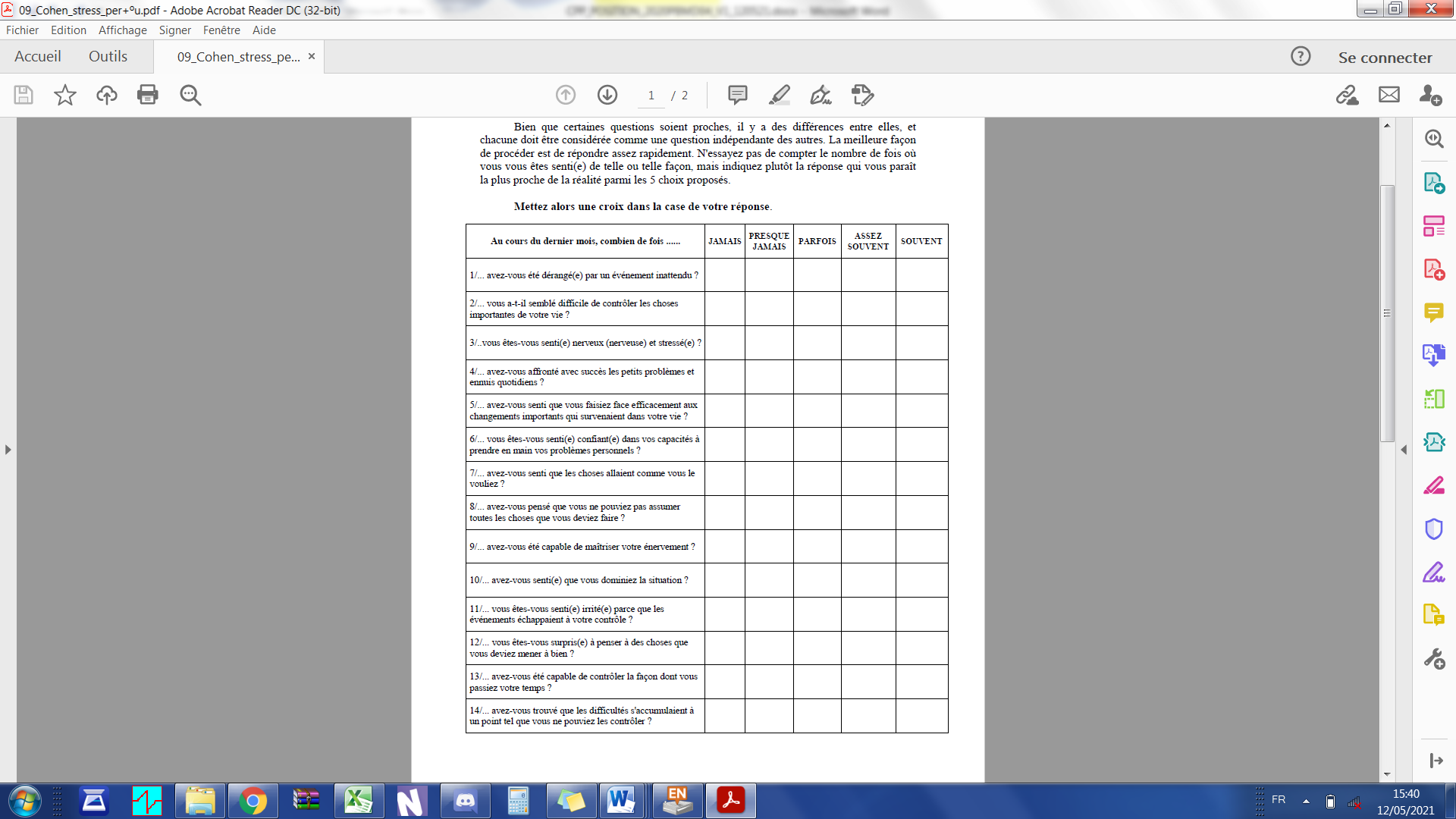
**

1. At the time the protocol was submitted, the Optimization of the Resources of the Armed Forces (ORAF) program was known as Potential Optimization Techniques (POT). [↑](#footnote-ref-1)
2. In the remainder of this document, and to improve readability, we use the term ‘fall injury’ to refer to an injury (or trauma) due to a fall resulting from a loss of balance. [↑](#footnote-ref-2)
3. ‘Advanced’ POTs training is defined as training that lasts longer than the initial training offered by the Ministry of the Armed Forces (10 hours). [↑](#footnote-ref-3)
4. All female candidates must provide evidence of a negative pregnancy test to take part in the selection course. [↑](#footnote-ref-4)
