# Supplementary material for: Position: A study protocol for the prevention of fall injuries in french special forces selection courses using a body-centered intervention
Source: PLoS One. 2023 Oct 4;18(10):e0290241. doi: 10.1371/journal.pone.0290241 (PMC10550174; doi:10.1371/journal.pone.0290241)
Supplement: S4 File — (DOCX) [file pone.0290241.s004.docx]

**PROTOCOLE RIPH2**

**PréventiOn de la bleSsure en milieu militaIre**

**par opTImisation de la cONscience corporelle**

**POSITION**

***

Version 1.0 du 21/07/2021

Référence du promoteur : 2020PBMD04 - Référence n° IDRCB : 2021-A02108-33

La recherche a reçu l'avis favorable du CPP NOM en date du jj/mm/aaaa

| **Investigateur coordonnateur/principal** | |
| --- | --- |
| Grade, Nom, Fonction : | Madame Anaïs DUFFAUD, investigateur coordonnateur |
| Etablissement : | Institut de Recherche Biomédicale des Armées |
| Adresse établissement : | 1, Place Général Valérie André, BP 73  91223 Brétigny-Sur-Orge |
| Téléphone : | 01-78-65-13-15 |
| E-mail : | anais.duffaud@def.gouv.fr |

| **Promoteur :** Direction Centrale du Service de Santé des Armées  Site de Vincennes, 60, boulevard du général Martial Valin CS 21623  75509 Paris Cedex 15 | |
| --- | --- |
| Contact : | Pharmacien chef des services Christophe Renard |
| Etablissement : | Direction de la Formation, de le Recherche et de l’Innovation - Bureau Recherche et Innovation |
| Adresse établissement : | DFRI Ilot Val-de-Grâce Bât. 8 3^ème^ étage 1 place Laveran  75230 Paris cedex 05 |
| Téléphone : | 01 40 51 41 03 |
| Fax : | 01 40 51 41 14 |
| E-mail : | dfri-bri.contact.fct@intradef.gouv.fr |

HISTORIQUE DES VERSIONS DU PROTOCOLE

*Section réservée au promoteur*

| **N° de version** | **Date** | **Motifs des modifications** |
| --- | --- | --- |
| 1.0 | 21/07/2021 | Création |
|  |  |  |
|  |  |  |
|  |  |  |

**PAGE DE SIGNATURE DU PROTOCOLE**

Prévention de la blessure en milieu militaire par optimisation de la conscience corporelle

POSITION

Ce protocole a été lu et approuvé à la date notée en en-tête.

Les deux parties s’engagent à mener la recherche conformément au protocole et aux dispositions législatives et réglementaires en vigueur.

**Investigateur coordonnateur**

Anaïs DUFFAUD, PhD

Unité neurophysiologie du stress

Département neurosciences et sciences cognitives

Institut de Recherche Biomédicale des Armées

**Promoteur**

Direction centrale du service de santé des armées

Représenté par Médecin Général Inspecteur Nathalie KOULMANN

Directeur de la Formation, de la Recherche et de l’Innovation

CE DOCUMENT CONFIDENTIEL EST LA PROPRIETE DU SERVICE DE SANTE DES ARMEES.

AUCUNE INFORMATION NON PUBLIEE FIGURANT DANS CE DOCUMENT NE PEUT ETRE DIVULGUEE SANS AUTORISATION ECRITE PREALABLE DE LA DIRECTION CENTRALE DU SERVICE DE SANTE DES ARMEES.

**SOMMAIRE**

[LISTE DES ABREVIATIONS 7](#_Toc78383345)

[RESUME DE LA RECHERCHE 8](#_Toc78383346)

[1. JUSTIFICATION SCIENTIFIQUE 11](#_Toc78383347)

[1.1. Etat actuel des connaissances 11](#_Toc78383348)

[1.1.1. Présentation de la pathologie / problème de santé et connaissances actuelles 11](#_Toc78383349)

[1.1.2. Connaissances actuelles sur les explorations prévues par le protocole 12](#_Toc78383350)

[1.2. Hypothèses de la recherche 15](#_Toc78383351)

[1.3. Retombées attendues 15](#_Toc78383352)

[2. OBJECTIFS 16](#_Toc78383353)

[2.1. Objectif principal 16](#_Toc78383354)

[2.2. Objectifs secondaires 16](#_Toc78383355)

[3. SCHEMA DE L’ETUDE 16](#_Toc78383356)

[3.1. Type d’essai 16](#_Toc78383357)

[3.2. Schéma d’étude 16](#_Toc78383358)

[3.2.1. Acte à l’étude 17](#_Toc78383359)

[3.2.2. Acte de comparaison 17](#_Toc78383360)

[3.2.3. Résumé 17](#_Toc78383361)

[3.3. Randomisation 18](#_Toc78383362)

[4. CRITERES D’ÉLIGIBILITE 18](#_Toc78383363)

[4.1. Modalités de recrutement 18](#_Toc78383364)

[4.2. Critères d’inclusion 18](#_Toc78383365)

[4.3. Critères de non inclusion 18](#_Toc78383366)

[5. CRITERES D’EVALUATION 19](#_Toc78383367)

[5.1. Critère d’évaluation principal 19](#_Toc78383368)

[5.2. Critères d’évaluation secondaires 19](#_Toc78383369)

[6. DEROULEMENT DE L’ETUDE 19](#_Toc78383370)

[6.1. Tableau récapitulatif du suivi patient 19](#_Toc78383371)

[6.2. Déroulement des visites liées à l’étude 20](#_Toc78383372)

[6.2.1. Visite d’inclusion 20](#_Toc78383373)

[6.2.2. Visite d’évaluation initiale 20](#_Toc78383374)

[6.2.3. Visites de suivi 20](#_Toc78383375)

[6.2.4. Visite de fin d’étude 21](#_Toc78383376)

[6.3. Fin de la recherche 21](#_Toc78383377)

[6.3.1. Critères d’arrêt de l’étude pour un sujet qui y participe 21](#_Toc78383378)

[6.3.2. Arrêt prématuré de la procédure expérimentale par le sujet 21](#_Toc78383379)

[6.3.3. Arrêt de l’étude par le promoteur 21](#_Toc78383380)

[6.3.4. Arrêt de l’étude par l’investigateur 21](#_Toc78383381)

[6.4. Contraintes liées à la recherche et indemnisation éventuelle des sujets 21](#_Toc78383382)

[6.5. Calendrier de la recherche 22](#_Toc78383383)

[7. DESCRIPTION DE L’ORGANISATION LOGISTIQUE DE L’ESSAI 22](#_Toc78383384)

[7.1. Logistique générale de la recherche 22](#_Toc78383385)

[7.2. Produits expérimentaux ou Dispositif médical expérimenté pour les besoins de l’étude 22](#_Toc78383386)

[8. VIGILANCE 22](#_Toc78383387)

[8.1. Définitions 22](#_Toc78383388)

[8.2. Méthodes et calendrier prévus pour l’évaluation de la sécurité 23](#_Toc78383389)

[8.3. Procédures de notification des effets indésirables, incidents ou risque d’incidents 23](#_Toc78383390)

[8.4. Modalités et durée de suivi des personnes suite à la survenue d’effets indésirables 24](#_Toc78383391)

[8.5. Effets indésirables graves tardifs 24](#_Toc78383392)

[9. COLLECTION D'ÉCHANTILLONS BIOLOGIQUES 24](#_Toc78383393)

[10. RECUEIL ET TRAITEMENT DES DONNÉES 24](#_Toc78383394)

[10.1. Description des données recueillies 24](#_Toc78383395)

[10.1.1. Paramètres cliniques 24](#_Toc78383396)

[10.1.2. Paramètres para-cliniques 25](#_Toc78383397)

[10.1.3. Paramètres biologiques 26](#_Toc78383398)

[10.2. Modalités de recueil des données 26](#_Toc78383399)

[10.3. Circuit des données 26](#_Toc78383400)

[10.4. Accès aux données et confidentialité 27](#_Toc78383401)

[11. ASPECTS STATISTIQUES 27](#_Toc78383402)

[11.1. Calcul du nombre de sujets nécessaire 27](#_Toc78383403)

[11.2. Méthodes statistiques employées 28](#_Toc78383404)

[12. CONTROLE ET ASSURANCE DE LA QUALITE 28](#_Toc78383405)

[12.1. Contrôle de qualité 29](#_Toc78383406)

[12.2. Audit et inspection 29](#_Toc78383407)

[12.3. Engagement de responsabilité 29](#_Toc78383408)

[13. CONSIDERATIONS ETHIQUES ET REGLEMENTAIRES 29](#_Toc78383409)

[13.1. Justification éthique du protocole 29](#_Toc78383410)

[13.1.1. Distinction soin / recherche 30](#_Toc78383411)

[13.1.2. Rapport bénéfices / risques 30](#_Toc78383412)

[13.1.2.1. Bénéfices 30](#_Toc78383413)

[13.1.2.2. Balance bénéfice/risque 30](#_Toc78383414)

[13.1.3. Choix méthodologiques 30](#_Toc78383415)

[13.2. Conformité aux textes de références 30](#_Toc78383416)

[13.3. Amendement au protocole 31](#_Toc78383417)

[14. CONSERVATION DES DOCUMENTS ET DES DONNEES RELATIVES A LA RECHERCHE 31](#_Toc78383418)

[15. REGLES RELATIVES A LA PUBLICATION 32](#_Toc78383419)

[15.1. Communications scientifiques 32](#_Toc78383420)

[15.2. Communication des résultats aux patients 32](#_Toc78383421)

[15.3. Cession des données 32](#_Toc78383422)

[16. REFERENCES 32](#_Toc78383423)

[17. ANNEXES 35](#_Toc78383424)

# LISTE DES ABREVIATIONS

ANSM Agence nationale de sécurité du médicament et des produits de santé

BPC Bonnes pratiques cliniques

BESS Balance error scoring system

CNIL Commission nationale de l’informatique et des libertés

CPP Comité de protection des personnes

CRF Case report form i.e. cahier d’observation

CV Curriculum vitae

DPD Délégué à la protection des données

EPMS Entrainement physique militaire et sportif

IRBA Institut de recherche biomédicale des armées

MR Méthodologie de référence

RPPS Répertoire partagé des professionnels de santé

SSA Service de santé des armées

STAC Stage de sélection des commandos marines

TOP Techniques d’optimisation du potentiel

# RESUME DE LA RECHERCHE

| Promoteur | Direction Centrale du Service de Santé des Armées |
| --- | --- |
| Personne qui dirige et surveille la recherche | Anaïs DUFFAUD, PhD  Unité neurophysiologie du stress, Département neurosciences et sciences cognitives, Institut de Recherche Biomédicale des Armées, BP 73, 91223 Brétigny-sur-Orge |
| Titre | Prévention de la blessure en milieu militaire par optimisation de la conscience corporelle - POSITION |
| Justification / contexte | L’activité physique et sportive joue un rôle important en milieu militaire pour développer et optimiser la capacité opérationnelle des combattants. Néanmoins, la prévalence des blessures est élevée et l’identification de facteurs de risques accessibles à des mesures de prévention primaire constitue un enjeu majeur pour la protection de la santé du combattant. Les données épidémiologiques militaires montrent que les blessures sont majoritairement des traumatismes aigus par chute suite à une perte d’équilibre. Ces données suggèrent l’hypothèse que la qualité de l’équilibre postural pourrait influencer la survenue des blessures rencontrées au cours des activités physiques militaires. Le niveau de stress élevé auxquels sont exposés les individus aux cours des activités physiques militaires pourrait contribuer à la dégradation de la qualité de l’équilibre postural. Le projet POSITION vise à étudier le mécanisme cognitif de conscience corporelle posturale comme facteur de risque de blessure, et comme cible d’une stratégie de prévention primaire basée sur les Techniques d’Optimisation du Potentiel (TOP). Les TOP pourraient améliorer le contrôle postural, et diminuer l’intensité de la réponse psychobiologique de stress, et ainsi réduire le taux d’incidence des blessures par chute survenant au cours des activités physiques militaires. |
| Objectifs | L’objectif principal sera d’évaluer l’efficacité des techniques d’optimisation du potentiel pour prévenir les blessures par chute au cours des activités physiques militaires.  Objectifs secondaires :  Le premier objectif secondaire sera de tester l’hypothèse mécanistique selon laquelle les TOP diminuent le risque de blessure par chute en améliorant le niveau de conscience corporelle posturale.  Le second objectif secondaire sera de tester l’hypothèse mécanistique selon laquelle les TOP diminuent le risque de blessure par chute en diminuant l’intensité de la réponse psychobiologique de stress au cours des activités physiques militaires.  Le troisième objectif secondaire sera de développer en machine learning un modèle prédictif du risque de blessure par chute à partir des mesures clinique (test BESS) et paraclinique (posturographie) effectuées avant le début des activités physiques militaires.  Le quatrième objectif secondaire sera d’évaluer l’effet de rémanence (persistance dans le temps) de l’efficacité d’une intervention TOP dans la prévention du risque de blessure par chute. Pour les participants qui poursuivront les activités physiques militaires après la visite de fin d’étude (et ce jusqu’à la fin du stage de sélection), nous comparerons le taux d’incidence des blessures par chute entre les groupes TOP et contrôle actif. |
| Schéma de la recherche | Nous réaliserons une étude de cohorte ouverte, prospective, contrôlée, monocentrique. |
| Critères d’évaluation | Le critère d’évaluation principal sera le taux d’incidence des blessures par chute suite à un déséquilibre dans chacun des deux groupes (TOP vs contrôle actif).  Critères de jugement secondaires :  Le premier objectif secondaire comprend deux critères d’évaluation : 1) le suivi du score à l’auto-questionnaire de conscience corporelle posturale (Postural Awareness Scale) entre avant et après l’intervention, ainsi qu’au début de chaque séance ; 2) la variation de la mesure de l’équilibre postural entre avant et après l’intervention.  Le second objectif secondaire comprend deux critères d’évaluation : 1) le suivi du score à l’auto-questionnaire de stress perçu de Cohen entre avant et après l’intervention, ainsi qu’au début de chaque séance ; 2) la variation de la charge allostasique dans la salive entre avant et après l’intervention (axe corticotrope : cortisol, DHEA, stress oxydatif ; système nerveux autonome : alpha amylase et chromogranine A).  Pour le troisième objectif secondaire, le critère d’évaluation sera une prédiction correcte du risque de blessure par chute à l’aide du modèle développé en machine learning.  Pour le quatrième objectif secondaire, le critère d’évaluation sera le taux d’incidence des blessures par chute suite à un déséquilibre dans chacun des deux groupes (TOP vs contrôle actif), dans la période suivant la fin de l’étude jusqu’à la fin du stage d’évaluation. |
| Critères d’inclusion | Signature d’un consentement éclairé de participation à l’étude  Participer au stage de sélection  Être un homme ou une femme âgée de plus de 18 ans  Être affilié à un régime de Sécurité Sociale |
| Critères de non inclusion | Participants ayant déjà bénéficié d’une formation « avancée » aux TOP (> 10 heures)  Refus de participer  Personne visée par les articles L1121-5 à L1121-8 du code de la santé publique, à savoir :  - femme enceinte, parturiente ou mère allaitante,  - personne privée de liberté par décision judiciaire ou administrative,  - personne faisant l’objet d’un suivi psychiatrique en vertu des articles L3112-1 et L3113-1 qui ne relèvent pas des dispositions de l’article L1121-8,  - personne majeure faisant l’objet d’une mesure de protection légale ou hors d’état d’exprimer leur consentement. |
| Déroulement de l’étude | L’étude comportera les visites suivantes :   - 1 visite d’inclusion : entretien d’inclusion et signature du consentement - 1 visite d’évaluation initiale: auto-questionnaires (médico-biographique, conscience corporelle posturale (Postural Awareness Scale) et niveau de stress (Cohen)), mesures clinique et paraclinique de la posture, et recueil de salive. - 4 visites de suivi réalisées au début des séances de l’intervention TOP ou contrôle actif et comprenant : auto-questionnaires (conscience corporelle posturale et niveau de stress), - 1 visite additionnelle sera effectuée pour les participants victimes d’un traumatisme par chute au cours du stage. Le participant complètera les questionnaires mesurant le niveau de conscience corporelle posturale et de stress perçu et une mesure de posturographie sera réalisée. - 1 visite de fin d’étude réalisée le jour suivant la dernière séance de l’intervention TOP ou contrôle actif, ou le jour de survenue de la blessure par chute si celle-ci survient avant la fin de l’intervention TOP ou contrôle actif. Elle comprendra: mesure paraclinique de la posture, auto-questionnaires (conscience corporelle posturale et niveau de stress) et recueil de salive |
| Nombre de sujets | L’échantillon devra inclure 200 participants pour chacun des deux bras (TOP et contrôle actif), soit au total 400 participants. |
| Analyse statistique | Pour l’objectif principal, nous évaluerons l’effet de l’intervention (TOP vs contrôle actif) sur le taux d’incidence des blessures par chute au moyen d’une analyse de survie comparative (modèle de Cox, avec les variables cliniques collectées comme facteurs confondants potentiels).  Pour le premier objectif secondaire, nous évaluerons l’effet de l’intervention TOP sur le niveau de conscience corporelle posturale en comparant dans le groupe TOP (i) les scores au questionnaire Postural Awareness Scale aux six temps de mesures (ANOVA à mesures répétées), et (ii) les variables de la posturographie avant et après l’intervention (tests de comparaison de moyennes pour échantillons appariés).  Pour le deuxième objectif secondaire, nous évaluerons l’effet de l’intervention TOP sur l’intensité de la réponse psychobiologique de stress en comparant dans le groupe TOP aux six temps de mesure (i) les scores au questionnaire de stress perçu de Cohen (ANOVA à mesures répétées), et (ii) les variables biologiques de stress (tests de comparaison de moyennes pour échantillons appariés).  Pour le troisième objectif secondaire, nous évaluerons en machine learning la performance du modèle prédictif du risque de blessure par chute en calculant la précision de la prédiction  Pour le quatrième objectif secondaire, nous évaluerons l’effet de rémanence de l’efficacité de l’intervention TOP sur prévention des blessures par chute, pendant la période entre la fin de l’étude et la fin du stage d’évaluation, au moyen d’une analyse de survie comparative (modèle de Cox). |
| Durée de la recherche | Durée de la période d’inclusion : 24 mois  Durée de l’intervention : 3 semaines  Durée de participation de chaque participant : 4 semaines  Durée totale de la recherche : 25 mois |

# JUSTIFICATION SCIENTIFIQUE

## Etat actuel des connaissances

### Présentation de la pathologie / problème de santé et connaissances actuelles

L’activité physique et sportive joue un rôle important en milieu militaire pour développer et optimiser la capacité opérationnelle des combattants. L’Entraînement Physique Militaire et Sportif (EPMS) vise à permettre à tout militaire d’acquérir les qualités physiques et mentales nécessaires à l’exercice de son métier (Etat-major des armées - Centre national des sports de la défense, 2011). De plus, la préservation de la capacité opérationnelle tout au long de la carrière militaire repose, entre autres, sur les bénéfices de la pratique sportive sur la santé, en particulier pour ce qui concerne la prévention des pathologies chroniques liées au stress (maladies cardio-vasculaires, dépression) (World Health Organization, 2003).

Néanmoins, l’activité physique et sportive est aussi la cause de pathologies musculo-squelettiques aigues (entorse, fracture, etc.) et chroniques (tendinopathies). En particulier, la prévalence des traumatismes des membres inférieurs est élevée au cours des activités physiques militaires, aussi bien chez les jeunes engagés volontaires [15-20%] (At, 2016; Bauvent, 2014; Pleche, 2018), qu’en unité militaire opérationnelle ou lors des épreuves de sélection des unités spécialisées [30-45%] (Bertrand, 2016; Brocard, 2014; Chipault, 2016; Longin, 2015; Morinière, 2013). Au-delà de leur conséquence sur la santé du combattant, ces blessures ont un impact négatif sur la capacité opérationnelle des forces (en raison des inaptitudes) et un coût économique non négligeable (Ressort, Desjeux, Marsan, & Thevenin-Garron, 2013).

Dans le cadre de l’élaboration d’une stratégie de prévention des blessures, identifier les facteurs de risque est une étape essentielle. Deux catégories de facteurs de risque sont classiquement décrits : (i) les facteurs de risque intrinsèques, spécifiques de l’individu, parmi lesquels l’âge, le genre, la condition physique ou la consommation de tabac ; et (ii) les facteurs de risque extrinsèques, qui se rapportent au contexte dans lequel l’activité sportive est effectuée, et parmi lesquels on retrouve les conditions environnementales (température, humidité, etc.), le type d’activité, ou les conditions d’entraînement (progressivité, régularité, etc.) (Bigard, Cravic, & Banzet, 2010; Meeuwisse, 1994; Williams, 1971). L’identification de facteurs de risques qui soient modifiables, i.e. accessibles à une intervention qui permettra de diminuer leur influence sur la survenue d’une blessure, reste un enjeu majeur dans le domaine du sport (Bahr & Holme, 2003).

Dans cette perspective, le projet de recherche POSITION vise à étudier le mécanisme cognitif de conscience corporelle comme facteur de risque de blessure par chute, et comme cible d’une stratégie de prévention ayant pour cible de réduire le taux d’incidence des blessures par chute en améliorant la qualité du contrôle postural.

Ce projet de recherche est né des observations épidémiologiques faites pendant le stage de sélection des commandos marines (STAC). L’inaptitude médicale déclarée en cours du stage à cause de la survenue d’une pathologie est la première cause d’échec au STAC (70%). Les pathologies sont majoritairement des traumatismes aigus (60%) principalement localisés aux membres inférieurs (Bertrand, 2016; Longin, 2015; Morinière, 2013). A la lecture des circonstances des traumatismes, une chute provoquée par une perte d’équilibre a été incriminée dans 90% des cas (Bertrand, 2016). Ces données suggèrent que la qualité de l’équilibre postural pourrait influencer la survenue des pathologies traumatiques rencontrées au cours du STAC. Les activités physiques militaires du STAC contraignent fortement l’équilibre postural : les stagiaires portent des charges lourdes (sac de combat avec arme, 15 kg) pendant des périodes prolongées, et réalisent des « parcours d’audace » situés en hauteur quelque soient les conditions météorologiques. Les données épidémiologiques recueillies au cours du STAC ont montré que la première activité pourvoyeuse de traumatismes est le parcours « jungle », parcours d’audace qui est réalisé sans assurance sur des obstacles situés jusqu’à 5 mètres de hauteur (Morinière, 2013). Des données épidémiologiques préliminaires (données non publiées) recueillies auprès d’autres unités militaires (Groupement d’Intervention de la Gendarmerie Nationale, Commando Parachutiste de l’Air n°10, 1er Régiment de Parachutistes d’Infanterie de Marine, 13ème Régiment de Dragons Parachutistes et Brigade des Sapeurs-Pompiers de Paris) semblent confirmer la forte fréquence des traumatismes en lien avec un déséquilibre postural au cours des activités physiques militaires.

Il convient d’ajouter que les activités physiques effectuées au sein des unités militaires suscitées se caractérisent par un très haut niveau de contraintes physiques et psychiques, qui mettent à rude épreuve l’organisme des individus et les exposent à un niveau de stress élevé. Les limites des individus sont poussées à l’extrême au moyen d’exercices physiques exigeants et intenses (chaque exercice étant réalisé en tenue de combat). A ces contraintes, s’ajoute la mise à l’épreuve psychique constante (stresseurs omniprésents : manque de sommeil, méconnaissance des déroulés des journées…) afin d’assurer la sélection d’impétrants aptes à réaliser les missions spécifiques aux unités militaires d’élite (Bertrand, 2016). L’ensemble de ces sollicitations vont activer la réponse neurobiologique de stress. Ce mécanisme biologique aspécifique, identique quel que soit l’agresseur (psychique ou physique), permet à l’organisme d’affronter les changements de son environnement qui portent atteinte à son intégrité, et de s’y adapter si ces changements perdurent. La qualité de cette réponse repose à la fois sur les caractéristiques du stresseur mais aussi sur celles de la qualité de la réactivité psychobiologique générale du sujet.

### Connaissances actuelles sur les explorations prévues par le protocole

Plusieurs systèmes neurophysiologiques contribuent à l’équilibre postural, et ce dernier ne se résume pas à une activité réflexe des muscles posturaux. L’équilibre postural met en jeu différents processus cognitifs de haut niveau comme l’attention, la perception visuo-spatiale et les fonctions exécutives (Amboni, Barone, & Hausdorff, 2013). L’information sensorielle issue des récepteurs périphériques (localisés dans les systèmes visuel, vestibulaire et somato-sensoriel) est intégrée au niveau du système nerveux central qui contrôle les muscles permettant d’orienter les différents parties du corps par rapport à la gravité (Forbes, Chen, & Blouin, 2018). Le mécanisme cognitif de conscience corporelle posturale se réfère à l’attention que porte l’individu à l’information qui est issue des systèmes somato-sensoriel et vestibulaire (Mehling et al., 2009). Plus l’individu prête attention à son corps, plus la quantité d’information disponible pour contrôler la posture est importante. Autrement dit, la qualité du contrôle postural pourrait être favorisée par un niveau élevé de conscience corporelle. Dans le cadre du projet de recherche POSITION, nous faisons l’hypothèse que le niveau individuel de conscience corporelle posturale pourrait influencer le risque de traumatisme par chute. En particulier, nous suggérons qu’un bas niveau de conscience corporelle posturale pourrait être un facteur de risque de traumatisme par chute, par l’intermédiaire d’un contrôle postural déficient. De plus, nous faisons l’hypothèse qu’une stratégie de prévention primaire des traumatismes par chute pourrait consister à améliorer le contrôle postural par l’augmentation du niveau de conscience corporelle posturale.

Il convient de noter que les systèmes neurophysiologiques impliqués dans le contrôle de la posture sont sous l’influence de structures cérébrales qui n’appartiennent pas à la boucle de contrôle postural. Ainsi, par exemple, le degré d’excitabilité du réflexe myotatique postural au niveau de la moelle épinière est modulé par des structures sous-corticales (notamment la substance grise périaqueducale et l’amygdale) qui sont activées au cours de la réaction de stress (Volchan et al., 2017). De façon intéressante, la conscience corporelle joue un rôle important dans la régulation de la réponse de stress en contribuant à l’identification, l’évaluation et la régulation de l’état physiologique interne du corps qui caractérise la réponse neurobiologique de stress (Craig, 2002). Ces différents éléments suggèrent que l’équilibre postural pourrait être modulé de manière indirecte par le mécanisme cognitif de conscience corporelle, via le contrôle de réponses non posturales (ex: la réponse de stress) qui impactent la posture. En résumé, le mécanisme de conscience corporelle pourrait influencer la survenue des blessures par chute soit (i) directement comme acteur central de la boucle de contrôle postural, ou (ii) indirectement en agissant sur des voies non posturales qui affectent la posture. La Figure 1 présente une synthèse du modèle théorique que nous testerons dans le cadre du projet POSITION.


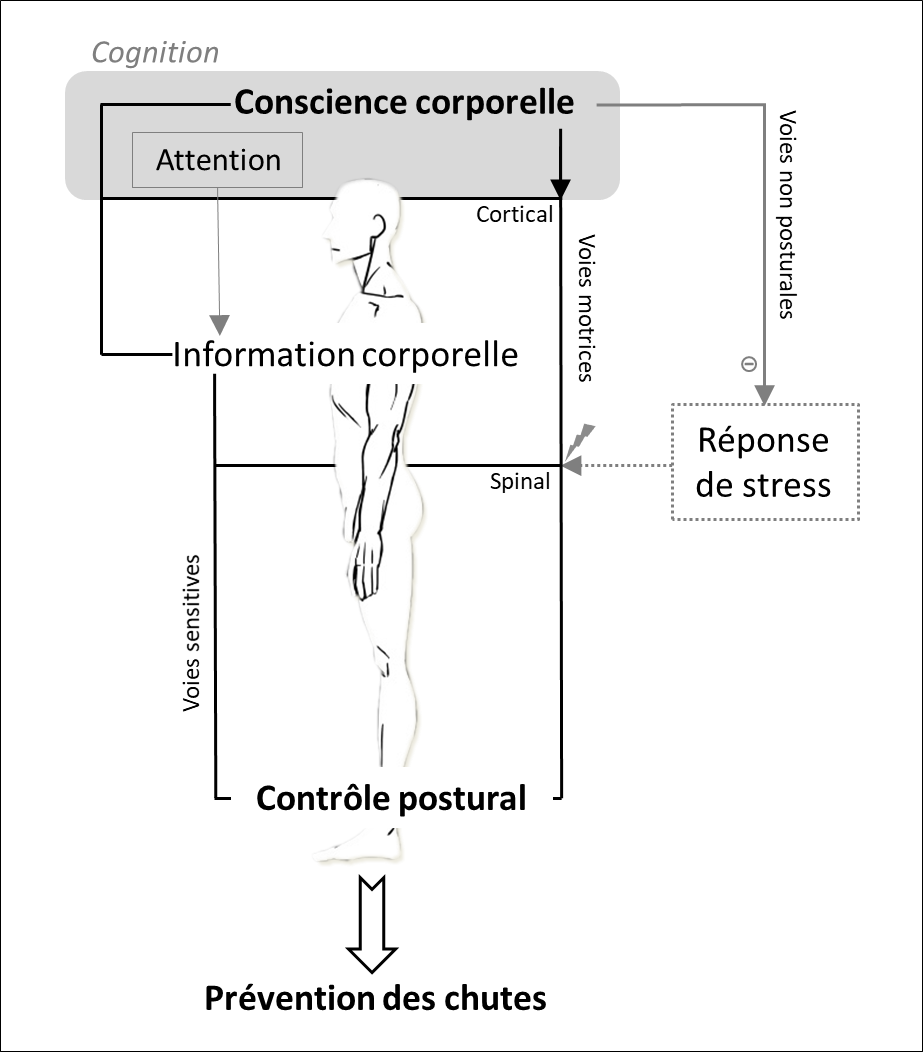


**Figure 1** - Modèle théorique selon lequel le mécanisme cognitif de conscience corporelle pourrait influencer la survenue des blessures par chute, soit *(i)* directement en agissant sur les voies motrices descendantes (flèche noire) car la conscience corporelle est un processus central dans la boucle de contrôle postural (lignes noires pleines), ou *(ii)* indirectement en contrôlant des voies non posturales (flèche grise) comme la réponse de stress qui altère la qualité du contrôle postural.

La posture en position debout est classiquement décrite selon le modèle biomécanique du « pendulum inversé » (Gage, Winter, Frank, & Adkin, 2004) : le corps se déplace en permanence le long d’un axe antéro-postérieur et d’un axe médio-latéral qui ont pour origine commune les malléoles internes des chevilles (Figure 2). La position debout est naturellement instable en raison de la physionomie du corps humain (les 2/3 de la masse corporelle sont situés sur les 2/3 supérieurs de la hauteur du corps) et des perturbations physiologiques endogènes (mouvements de la respiration, battements cardiaques, etc.). Le contrôle postural est donc continuellement mis en œuvre pour maintenir le corps autour d’un point d’équilibre, et ainsi éviter la chute.


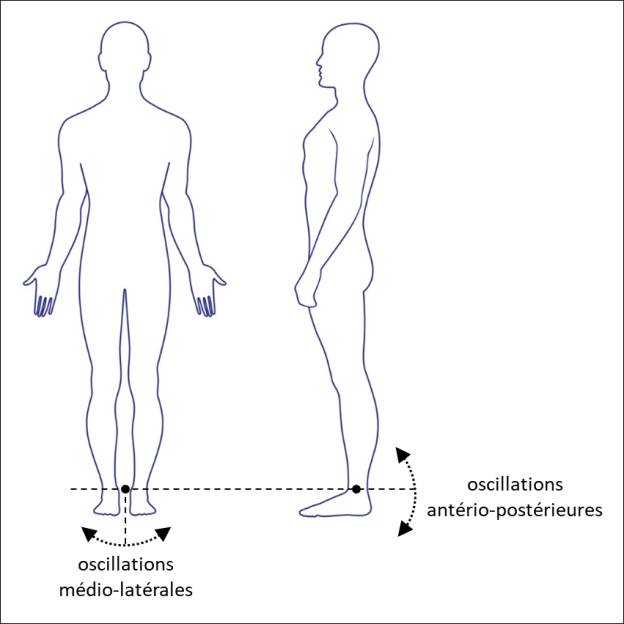


**Figure 2** – Modèle biomécanique de l’équilibre postural statique, appelé aussi modèle du « pendulum inversé ». Le corps se déplace en permanence le long d’un axe antéro-postérieur et d’un axe médio-latéral qui ont pour origine commune les malléoles internes des chevilles.

En médecine du sport, l’équilibre postural statique est le plus souvent évalué par le test Balance Error Scoring System (BESS) (Delahunt et al., 2018; Docherty, Valovich McLeod, & Shultz, 2006). Ce test clinique doit être effectué par un médecin et dure environ cinq minutes. Dans le domaine des neurosciences cognitives, un travail récent a montré l’intérêt d’un outil paraclinique, la posturographie, pour évaluer l’équilibre postural et rendre compte du niveau de conscience corporelle des individus (C. Verdonk, Trousselard, Medani, Vialatte, & Dreyfus, 2020). Les outils d’analyse développés dans ce travail ont été utilisés ensuite pour développer un outil d’évaluation du risque de blessure par chute au cours du STAC. Les analyses préliminaires montrent que l’analyse de la posturographie permet l’évaluation correcte du risque de blessure chez 75% des stagiaires commando marines (C. Verdonk et al., 2019).

Le projet de recherche POSITION s’intéresse au mécanisme cognitif de conscience corporelle comme facteur de risque de blessure par chute au cours des activités physiques militaires. Dans ce cadre, il est indispensable d’évaluer le caractère modifiable du facteur de risque afin de pouvoir envisager le développement d’une stratégie de prévention des blessures. Le caractère modifiable signifie que le facteur de risque est accessible à une intervention qui permettra de contrôler son influence sur la survenue de la blessure (Bahr & Holme, 2003). Dans le cadre du projet de recherche POSITION, nous testerons l’effet des Techniques d’Optimisation du Potentiel (TOP) sur le mécanisme cognitif de conscience corporelle. Nous faisons l’hypothèse que la pratique des TOP augmenterait le niveau de conscience corporelle, et ainsi diminuer le risque de blessure par chute grâce à une amélioration du contrôle postural.

Les TOP ont vu le jour dans les années 90 grâce au Docteur Edith Perreault-Pierre pour répondre à des besoins, qui jusqu’alors, n’avaient jamais été abordés dans les armées : la préparation mentale et la récupération (Etat-major des armées - Centre national des sports de la défense, 2011). Initialement développés pour répondre aux attentes de l’armée de l’air en termes de gestion du stress et de prise en compte du facteur humain (dans le cadre de la sécurité des vols), ils sont maintenant déployés dans l’ensemble des autres corps de l’Armée.

Les TOP se définissent comme une méthode de préparation mentale qui vise l’apprentissage d’un ensemble de techniques et de stratégies mentales permettant de mobiliser au mieux les ressources physiques et psychologiques d’un individu, en fonction des exigences des situations qu’il rencontre (Figure 3).


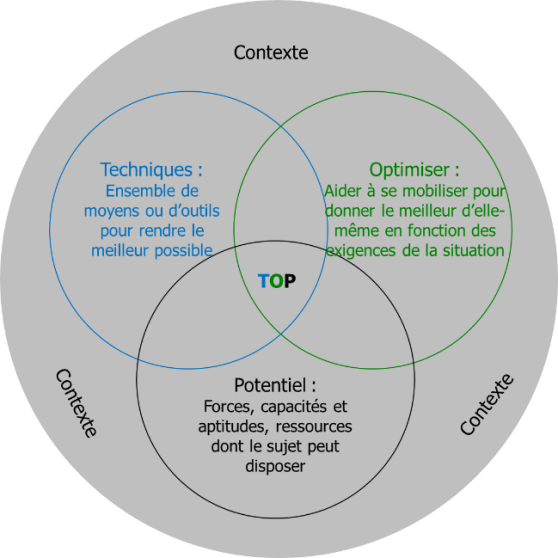


**Figure 3** – Les 3 composantes des Techniques d’Optimisation du Potentiel (TOP).

Les techniques utilisées font appel à des procédés de base connus du sport de haut niveau. La méthode se présente sous la forme d’une « boîte à outils » personnalisable qui doit permettre aux personnels formés de mener à bien leurs missions tout en prévenant les effets délétères du stress. Les effets des techniques d’imagerie mentale, de dialogue interne, de relaxation et de « pleine conscience » sur la performance mais aussi la santé et le bien-être sont très bien décrits dans la littérature (Pagninia, Manzonib, Castelnuovoab, & Molinariab, 2013; Vealey & Greenleaf, 2010; William, Zinsser, & Bunker, 2010). Les données disponibles sur les TOP sont, en revanche, parcellaires. Le Tableau en ANNEXE 1, présente un résumé synthétique des études ayant évalué les effets d’une formation TOP sur les performances psycho-cognitives et physiques, sur le stress et sur le sommeil. Bien que les niveaux de preuves de ces études ne soient pas tous équivalents (plusieurs études interventionnelles avant-après et un essai clinique randomisé contrôlé), l’ensemble de ces résultats apporte un faisceau d’arguments en faveur de bénéfices cliniques et opérationnels des TOP.

## Hypothèses de la recherche

Le projet de recherche POSITION a pour but d’évaluer l’efficacité des TOP sur la prévention des blessures par chute suite à un déséquilibre^[[1]](#footnote-1)^. Nous émettons l’hypothèse que les TOP pourraient diminuer le risque de blessure par chute en améliorant la qualité du contrôle postural par l’intermédiaire d’une augmentation du niveau de conscience corporelle posturale.

## Retombées attendues

Les activités physiques militaires sont associées à une forte prévalence des blessures par chute. Un enjeu majeur pour la médecine militaire est d’identifier les individus à risque de blessure par chute, afin de pouvoir leur proposer des contre-mesures préventives personnalisées visant à diminuer le risque de blessure. Ceci implique de développer un outil d’évaluation du risque de blessure, sur la base de l’évaluation d’un facteur de risque qui soit par ailleurs accessible à une stratégie de prévention permettant de diminuer son influence sur la survenue d’une blessure. Une des retombées du projet POSITION sera le développement d’un outil permettant d’identifier les individus à risque de blessure par chute à partir de l’analyse de l’équilibre postural (le test clinique BESS et la posturographie).

De plus, l’exploration du mécanisme cognitif de conscience corporelle posturale permettra de mieux comprendre la variabilité inter-individuelle face au risque de blessure par chute pendant les activités physiques militaires.

Enfin, l’évaluation du bénéfice des TOP sur la diminution du risque de blessure permettra d’apprécier l’intérêt de ce programme de prévention, déjà en place dans les armées, pour la prévention des traumatismes par chute.

# OBJECTIFS

## Objectif principal

L’objectif principal sera d’évaluer l’efficacité des techniques d’optimisation du potentiel pour prévenir les blessures par chute au cours des activités physiques militaires. Pour cela, nous analyserons l’effet d’une intervention TOP sur le taux d’incidence des blessures par chute survenant au cours des activités physiques militaires, par comparaison à une intervention contrôle actif.

## Objectifs secondaires

L’étude aura 4 objectifs secondaires :

1. Le premier objectif secondaire sera de tester l’hypothèse mécanistique selon laquelle les TOP diminuent le risque de blessure par chute en améliorant le niveau de conscience corporelle posturale.
2. Le second objectif secondaire sera de tester l’hypothèse mécanistique selon laquelle les TOP diminuent le risque de blessure par chute en diminuant l’intensité de la réponse psychobiologique de stress au cours des activités physiques militaires.
3. Le troisième objectif secondaire sera de développer en machine learning un modèle prédictif du risque de blessure par chute à partir des mesures clinique (test BESS) et paraclinique (posturographie) effectuées avant le début des activités physiques militaires.
4. Le quatrième objectif secondaire sera d’évaluer l’effet de rémanence (persistance dans le temps) de l’efficacité d’une intervention TOP dans la prévention du risque de blessure par chute. Pour les participants qui poursuivront les activités physiques militaires après la visite de fin d’étude (et ce jusqu’à la fin du stage de sélection), nous comparerons le taux d’incidence des blessures par chute entre les groupes TOP et contrôle actif.

# SCHEMA DE L’ETUDE

## Type d’essai

Cette étude rentre dans le cadre des recherches impliquant la personne humaine de type interventionnelle à risques et contraintes minimes, car d’une part elle propose l’évaluation d’une intervention TOP d’usage au sein du Ministère des armées, et elle implique d’autre part le recueil de données biomédicales (physiologique et psychologique) à caractère non-invasif et qui ne renseignent pas sur la nature pathologique de l’état de santé de l’individu.

Pour répondre aux différents objectifs de ce projet de recherche, nous réaliserons une étude de cohorte prospective, contrôlée. Cette étude ouverte sera conduite sur des individus volontaires sains avec bénéfice direct. Cette étude s’intégrera dans le cadre de l’activité de soutien sanitaire qui est habituellement mise en place au cours des activités physiques militaires.

## Schéma d’étude

Afin de garantir le respect du protocole expérimental pendant les deux années que durera l’étude (voir le paragraphe 3.3 pour une explication détaillée), l’intervention contrôle actif sera faite la première année de l’étude (année A), tandis que l’intervention TOP sera faite la deuxième année de l’étude (année A+1).

L’étude se déroulera sur six unités militaires :

1. Groupement d’Intervention de la Gendarmerie Nationale (Versailles-Satory, 78)
2. Commando Parachutiste de l’Air n°10 (Orléans, 45)
3. 1er Régiment de Parachutistes d’Infanterie de Marine (Bayonne, 64)
4. 13ème Régiment de Dragons Parachutistes (Souge, 33)
5. Groupement Formation Instruction et de Secours de la Brigade des Sapeurs-Pompiers de Paris (Villeneuve-Saint-Georges, 94)
6. Ecole des fusiliers marins et commandos (Lorient, 56)

L’étude se déroulera pendant les stages d’évaluation qui sont organisés dans chaque unité, et la durée de l’étude sera de trois semaines.

### Acte à l’étude

L’intervention TOP sera constituée d’un enseignement pratique des TOP dispensé par le moniteur TOP identifié au sein de chaque unité militaire, et qui est un personnel militaire ayant bénéficié de la formation Moniteur TOP délivrée par le Centre National des Sports de Défense. L’intervention se déroulera sur six séances d’une heure, réparties tout au long des trois semaines de l’étude à une fréquence de deux séances par semaine. Les deux premières séances de l’intervention TOP seront programmées au début du stage d’évaluation (idéalement la veille du démarrage de celui-ci)

L’intervention inclura un enseignement théorique et la pratique des techniques TOP qui ont été identifiées comme pertinentes pour augmenter le niveau de conscience corporelle (attention portée sur la respiration, relaxation musculaire, imagerie mentale ; voir ANNEXE 2 pour le descriptif du contenu de l’intervention TOP). Le contenu des six séances de l’intervention TOP a été préalablement défini et validé par les moniteurs TOP des six unités militaires, sous la supervision de la monitrice TOP coordinatrice de l’intervention.

### Acte de comparaison

L’intervention contrôle actif se déroulera selon les mêmes modalités que l’intervention TOP : six séances d’une heure, réparties tout au long des trois semaines de l’étude à une fréquence de deux séances par semaine. Lors de ces séances, des activités cognitives seront proposées (le détail des séances est disponible en ANNEXE 3) pour travailler les processus cognitifs de haut niveau tels que le raisonnement, le traitement de l’information, ou encore la créativité. Les séances s’articuleront en deux temps : tout d’abord un volet théorique pour décrire le processus travaillé lors de la séance, puis un volet pratique avec des exercices de mise en situation.

Ces séances seront réalisées sous la supervision du personnel en charge de l’intervention TOP l’année suivante (moniteur TOP).

### Résumé


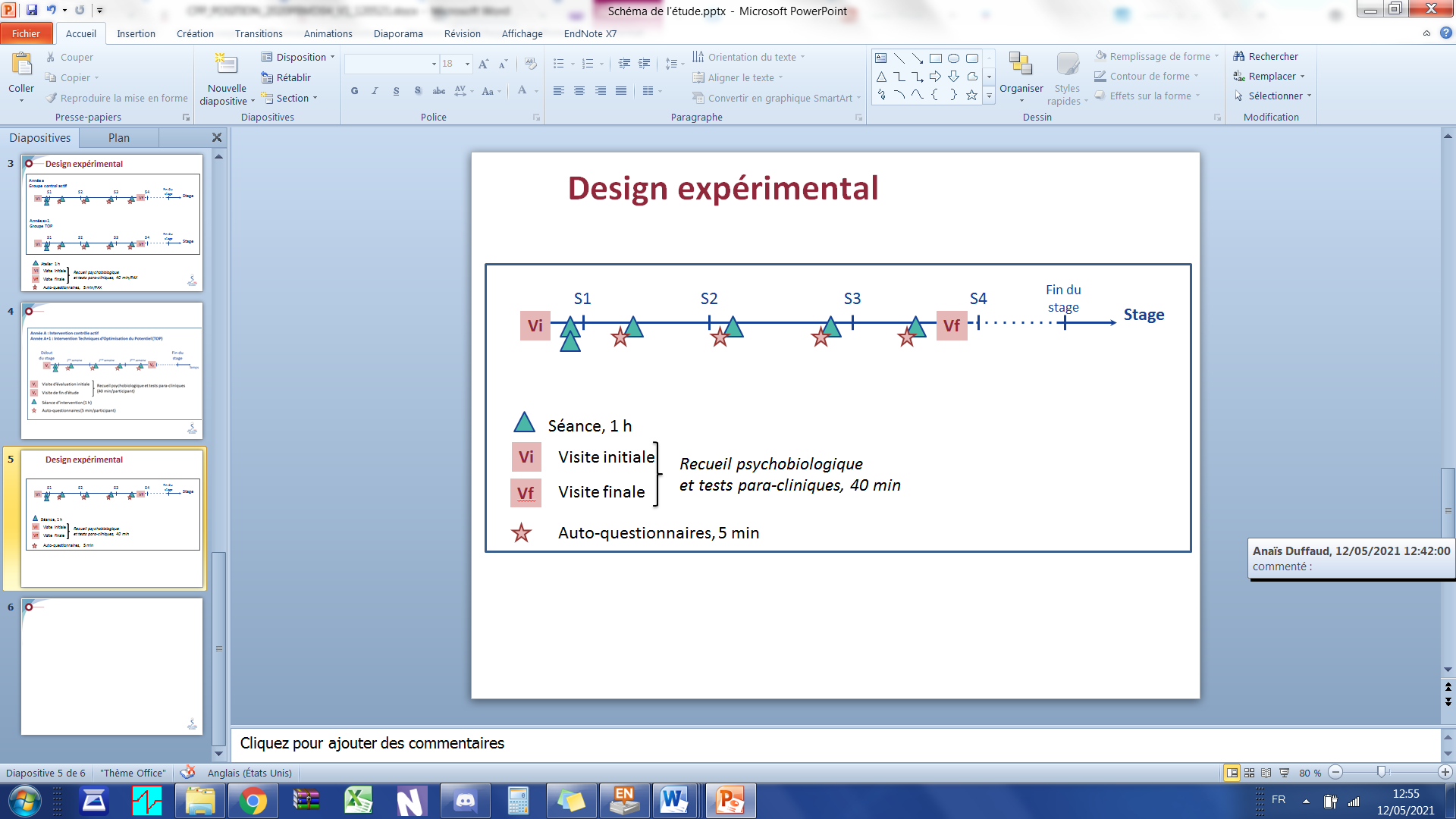


**Figure 4** - Schéma de l’étude pour une année considérée. Le déroulé de l’étude sera identique pour les deux années que durera l’étude, hormis la nature des séances qui seront du contrôle actif l’année A et des TOP l’année A+1.

## Randomisation

L’intervention (TOP versus contrôle actif) ne sera pas randomisée de façon individuelle (pour chaque participant dans chaque unité militaire). En effet, cette randomisation au sein de chaque unité n’est pas possible en raison du risque d’influence des participants du groupe TOP et ceux du groupe témoin actif (de biais de « contamination »). Les conditions de réalisation du stage d’évaluation impliquent une forte promiscuité entre les participants des deux groupes. D’autre part, la différence d’intervention au sein de chaque stage risque de déstabiliser l’effet souhaité de cohésion d’équipe. C’est pourquoi nous avons choisi de réaliser l’étude sur deux années successives (années A et A+1) :

1. Les participants inclus au cours de l’année A bénéficieront de l’intervention contrôle actif
2. Les participants inclus au cours de l’année A+1 bénéficieront de l’intervention TOP.

L’ordre des interventions (contrôle actif puis TOP) n’est volontairement pas randomisé entre les unités afin que le protocole de recherche soit respecté pendant toute la durée de l’étude. En effet, si un effet bénéfique de l’intervention TOP était observé à l’année A, il serait très difficile de recueillir l’accord des différentes unités pour mettre en place une intervention contrôle actif à l’année A+1. Par ailleurs, il serait sans doute difficile aux unités de ne pas réaliser d’action ayant montré un éventuel bénéfice auprès des candidats.

# CRITERES D’ÉLIGIBILITE

## Modalités de recrutement

Les participants seront des volontaires recrutés parmi les candidats au stage d’évaluation organisé dans les différentes unités. Les participants seront des sujets en bonne santé et en bonne condition physique, car leur profil médical aura été déclaré conforme au profil médical d’aptitude exigé pour l’inscription au stage d’évaluation. La participation à l’étude sera proposée à tous les candidats au stage d’évaluation.

## Critères d’inclusion

- Signature d’un consentement éclairé de participation à l’étude
- Participer au stage de sélection
- Être un homme ou une femme âgée de plus de 18 ans
- Être affilié à un régime de Sécurité Sociale

## Critères de non inclusion

- Participants ayant déjà bénéficié d’une formation « avancée » aux TOP (> 10 heures)^[[2]](#footnote-2)^
- Refus de participer
- Personne visée par les articles L1121-5 à L1121-8 du code de la santé publique, à savoir
  - femme enceinte^[[3]](#footnote-3)^, parturiente ou mère allaitante,
  - personne privée de liberté par décision judiciaire ou administrative,
  - personne faisant l’objet d’un suivi psychiatrique en vertu des articles L3112-1 et L3113-1 qui ne relèvent pas des dispositions de l’article L1121-8,
  - personne majeure faisant l’objet d’une mesure de protection légale ou hors d’état d’exprimer leur consentement.

# CRITERES D’EVALUATION

## Critère d’évaluation principal

Le critère d’évaluation principal sera la fréquence des blessures par chute suite à un déséquilibre dans chacun des deux groupes (TOP *vs* contrôle actif).

L’évènement de blessure par chute sera enregistré par le médecin militaire en charge du soutien sanitaire des activités physiques militaires. Pour le participant blessé, la survenue de l’évènement déclenchera la réalisation d’une visite de suivi additionnelle le même jour après la consultation médicale.

## Critères d’évaluation secondaires

Les critères permettant de répondre aux objectifs secondaires seront :

- - 1. Le premier objectif secondaire comprend deux critères d’évaluation : 1) le suivi du score à l’auto-questionnaire de conscience corporelle posturale (Postural Awareness Scale, ANNEXE 4) entre avant et après l’intervention, ainsi qu’au début de chaque séance ; 2) la variation de la mesure de l’équilibre postural entre avant et après l’intervention.
    2. Le second objectif secondaire comprend deux critères d’évaluation: 1) le suivi du score à l’auto-questionnaire de stress perçu de Cohen (ANNEXE 4) entre avant et après l’intervention, ainsi qu’au début de chaque séance; 2) la variation de la charge allostasique dans la salive entre avant et après l’intervention (axe corticotrope : cortisol, DHEA, stress oxydatif; système nerveux autonome : alpha amylase et chromogranine A).
    3. Pour le troisième objectif secondaire, le critère d’évaluation sera une prédiction correcte du risque de blessure par chute à l’aide du modèle développé en machine learning.
    4. Pour le quatrième objectif secondaire, le critère d’évaluation sera le taux d’incidence des blessures par chute dans chacun des deux groupes (TOP *vs* contrôle actif), dans la période suivant la fin de l’étude jusqu’à la fin du stage d’évaluation. Les informations concernant la fin de stage de chaque participant seront recueillies auprès du médecin responsable du stage.

# DEROULEMENT DE L’ETUDE

Au cours du déroulement de la recherche, les participants seront vus dans les lieux suivants :

L’ensemble des mesures effectuées seront réalisées sur le site où se déroule le stage de sélection de l’unité militaire impliquée. L’inclusion et la visite de fin d’étude seront réalisées au sein de l’antenne médicale de rattachement de l’unité militaire. Les visites de suivi tout comme les séances TOP ou séances contrôle actif, seront conduites sur le terrain même où le stage se déroulera.

## Tableau récapitulatif du suivi patient

**Tableau 1** - Récapitulatif des visites liées à l’étude


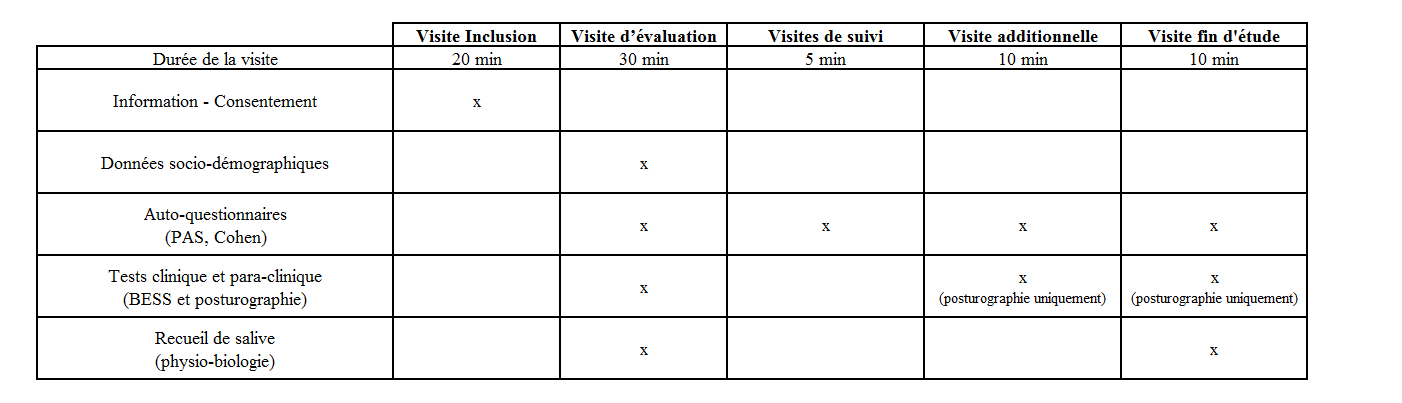


## Déroulement des visites liées à l’étude

La veille du démarrage du stage de sélection, les candidats recevront une information collective par l'investigateur ou un de ses collaborateurs et une lettre d’information sur l’étude. Les candidats bénéficieront d’un délai de réflexion de plusieurs heures avant la visite d’inclusion.

### Visite d’inclusion

Lors de la visite d’inclusion qui se déroulera le lendemain de l’information collective et avant tout examen lié à la recherche, l'investigateur ou un de ses collaborateurs proposera au participant de participer à cette recherche et l’informera :

- de l’objectif, la nature des contraintes,
- les risques prévisibles et les bénéfices attendus de la recherche
- du traitement informatisé des données le concernant qui seront recueillies au cours de cette recherche et lui précise également ses droits d’accès, d’opposition et de rectification à ces données,
- de la conservation à des fins scientifiques de ses prélèvements à l’issue de la recherche et recueillera sa non-opposition.

L’investigateur ou un de ses collaborateurs répondra ensuite à toutes les questions du participant. Ce dernier disposera alors d’un délai de réflexion de 15 minutes avant de prendre sa décision concernant sa participation à l’étude. Celle-ci sera formalisée par le signature du document de consentement (en 3 exemplaires).

Tout amendement qui modifie la prise en charge des patients ou les bénéfices, risques et contraintes de la recherche fera l’objet d’un nouveau document d’information. L’information des personnes concernées suivra la même procédure.

Les différents exemplaires de la note d’information et du formulaire de consentement seront alors répartis comme suit :

- Un exemplaire de la note d'information et du consentement signé sera remis au sujet.
- L’exemplaire original sera conservé par le médecin investigateur dans un lieu sûr inaccessible à des tiers.

A la fin des inclusions ou au plus tard à la fin de la recherche, une copie de chaque formulaire de consentement sera transmise au promoteur ou à son représentant selon les modalités communiquées.

### Visite d’évaluation initiale

Pour les sujets ayant donné leur consentement pour participer à l’étude, la visite d’évaluation initiale aura lieu immédiatement après la visite d’inclusion. L’investigateur ou l’un de ses collaborateurs recueillera les données cliniques (âge, taille, poids et genre), et effectuera le test clinique BESS ainsi que la mesure de posturographie. Le participant complètera ensuite un questionnaire médico-démographique, ainsi que les questionnaires permettant la mesure du niveau de conscience corporelle posturale (Postural Awareness Scale) et de stress perçu (Cohen). Enfin, le recueil d’un échantillon de salive de 4 ml sera effectué.

### Visites de suivi

Une visite de suivi sera réalisée juste avant chacune des séances des interventions TOP et contrôle actif (dont le calendrier est schématisé en Figure 4). La visite de suivi consistera pour le participant à remplir les questionnaires mesurant le niveau de conscience corporelle posturale (Postural Awareness Scale) et de stress perçu (Cohen).

Une visite de suivi additionnelle sera effectuée pour les participants victimes d’un traumatisme par chute au cours du stage. Cette visite aura lieu le jour de survenue du traumatisme. Le médecin militaire ou un de ses collaborateurs recueillera les informations médicales relatives au traumatisme et effectuera une mesure de posturographie. Le participant complètera ensuite les questionnaires mesurant le niveau de conscience corporelle posturale (Postural Awareness Scale) et de stress perçu (Cohen).

### Visite de fin d’étude

Le suivi du participant prendra fin (i) soit le jour suivant la dernière séance de l’intervention TOP ou contrôle actif, (ii) soit le jour de la sortie du stage (quel qu'en soit la cause) si celle-ci intervient avant la fin de l’intervention (TOP ou contrôle actif).

Le médecin militaire ou l’un de ses collaborateurs effectuera une mesure de posturographie et le participant complètera les questionnaires mesurant le niveau de conscience corporelle posturale (Postural Awareness Scale) et de stress perçu (Cohen). Enfin, le recueil d’un échantillon de salive de 4 ml sera effectué.

## Fin de la recherche

### Critères d’arrêt de l’étude pour un sujet qui y participe

La participation d’un sujet prend fin :

- A la dernière séance de l’intervention (TOP ou contrôle actif),
- Lors de la sortie du stage (quel qu'en soit la cause) si celle-ci intervient avant la fin de l’intervention.

Tout arrêt de l’étude pour un sujet qui y participe sera définitif.

Aucun critère d’exclusion n’est prévu au protocole.

### Arrêt prématuré de la procédure expérimentale par le sujet

Tout sujet pourra arrêter sa participation à la recherche, à n’importe quel moment et quelle qu’en soit la raison. En cas d’arrêt prématuré de la participation à la recherche, les données recueillies jusqu’à la date de retrait du consentement le concernant pourront être utilisées, sauf si le participant s’y oppose, conformément à l’article L 1122-1-1 du code de la santé publique. Les participants ne seront pas remplacés

### Arrêt de l’étude par le promoteur

Le promoteur pourra arrêter l'étude à tout moment, pour les raisons suivantes :

- Incapacité de l'investigateur à inclure les sujets selon le calendrier prévu.
- Absence de consentement signé.
- Violations majeures au protocole.
- Données incomplètes ou erronées.

Le promoteur établira une déclaration de fin d’essai dans un délai de 90 jours suivant la fin de la recherche.

Si l’essai clinique est arrêté (définitivement) de façon anticipée, cet arrêt devra être déclaré à l’ANSM et au CPP dans un délai de 15 jours en indiquant les raisons qui le motivent.

### Arrêt de l’étude par l’investigateur

En cas d'événement indésirable jugé sévère par l'investigateur et pouvant mettre en jeu la santé des sujets, l'investigateur pourra arrêter l'étude en accord avec le promoteur.

## Contraintes liées à la recherche et indemnisation éventuelle des sujets

Les participants ne pourront pas prendre simultanément part à un autre projet de recherche. Aucune indemnisation n’est prévue.

## Calendrier de la recherche

- Durée de la période d’inclusion : 24 mois
- Durée de l’intervention : 3 semaines
- Durée de participation de chaque participant : 4 semaines
- Durée totale de la recherche : 25 mois

# DESCRIPTION DE L’ORGANISATION LOGISTIQUE DE L’ESSAI

## Logistique générale de la recherche

L’investigatrice coordinatrice, avec l’aide de son collaborateur scientifique, supervisera la réalisation de l’ensemble des visites de l’étude (voir paragraphe 6.2). Pour les visites d’inclusion et d’évaluation initiale, l’investigatrice coordinatrice et son collaborateur scientifique seront aidés par le personnel soignant (médecins, infirmiers et brancardiers-secouristes) des unités militaires dans lesquels se déroule l’étude. Les visites de suivi effectuées avant chaque séance de l’intervention (TOP ou contrôle actif) seront menées par le moniteur TOP. La visite de suivi additionnelle (réalisée si le participant est victime d’un traumatisme par chute pendant la durée de l’étude) ainsi que la visite de fin d’étude seront menées par le médecin militaire de l’untié ou un de ses collaborateurs.

L’investigatrice coordinatrice sera aussi chargée de la surveillance de la recherche, de l’établissement des rapports concernant son état d’avancement et de la vérification de la mise à jour du cahier d’observation (demande d’informations complémentaires, corrections, etc.).

## Produits expérimentaux ou Dispositif médical expérimenté pour les besoins de l’étude

Aucun produit expérimental ne sera administré aux participants, et aucun dispositif médical ne sera expérimenté dans le cadre de cette recherche.

# VIGILANCE

## Définitions

Un **événement indésirable** est une manifestation nocive survenant chez une personne qui se prête à une recherche interventionnelle, que cette manifestation soit liée ou non à la recherche ou au produit sur lequel porte cette recherche.

Un **effet indésirable** est un événement indésirable pour lequel un lien de causalité (même faible) est suspecté ou établi avec un produit de santé ou un acte médical.

Un **effet indésirable grave (EIG)** se définit comme tout événement ou effet indésirable qui entraîne la mort, met en danger la vie de la personne qui se prête à la recherche, nécessite une hospitalisation ou la prolongation de l'hospitalisation, provoque une incapacité ou un handicap importants ou durables, ou bien se traduit par une anomalie ou une malformation congénitale, et s'agissant du médicament, quelle que soit la dose administrée.

Un i**ncident** ou un **risque d’incident grave** correspond à la mise en cause d’un dispositif médical dans un évènement ayant entraîné ou ayant été susceptible d’entraîner la mort ou la dégradation grave de l’état de santé d’un patient, d’un utilisateur ou d’un tiers.

Un **fait nouveau** est toute nouvelle donnée pouvant conduire à une réévaluation du rapport des bénéfices et des risques de la recherche ou du produit objet de la recherche, à des modifications dans l’utilisation de ce produit, dans la conduite de la recherche, ou des documents relatifs à la recherche, ou à suspendre ou interrompre ou modifier le protocole de la recherche ou des recherches similaires. Dans le cas des essais portant sur la première administration à l’homme ou sur l’utilisation d’un produit de santé chez des personnes ne présentant aucune affection, tout effet indésirable grave est un fait nouveau.

## Méthodes et calendrier prévus pour l’évaluation de la sécurité

Aucun paramètre spécifique ne sera mis en place pour le suivi de la sécurité des patients. Les investigateurs devront déclarer selon les procédures habituelles entrant dans le cadre du soin les effets indésirables ; incidents et risques d’incidents graves qu’ils auront identifiés ou suspectés pendant toute la durée de participation du patient à l’étude.

## Procédures de notification des effets indésirables, incidents ou risque d’incidents

Dès qu’un investigateur prendra connaissance d’un effet indésirable, d’un incident ou risque d’incident grave, il le notifiera directement à la vigilance concernée (Tableau ci-dessous) selon les procédures en vigueur dans l’établissement et en précisant dans la déclaration que le patient est inclus dans l’étude POSITION.

| Evènement lié à : | Vigilance sanitaire ou agence concernée | Structure concernée |
| --- | --- | --- |
| Un médicament | Pharmacovigilance | Centre Régional de Pharmacovigilance (CRPV) |
| Un produit sanguin | Hémovigilance | Correspondant local d’hémovigilance (CRH) |
| Un dispositif médical | Matériovigilance | Correspondant local de matériovigilance |
| Un acte lié au(x) soin(s) | Pas de vigilance sanitaire. Transmission directe à -> | Agence Régionale de Santé (ARS)* |
| Une infection liée au(x) soin(s) | Infectiovigilance | Correspondant local d’infectiovigilance |
| Une substance toxique non médicamenteuse | Toxicovigilance | Centre Antipoison (CAP-TV) |
| Une substance psychogène avec conduite addictive ou pharmacodépendance | Addictovigilance | Centre d’Evaluation et d’Information sur les pharmacodépendances-addictovigilance (CEIP-A) |
| Un aliment, un complément alimentaire, un produit falsifié | Toxicovigilance | Centre Antipoison (CAP-TV) |

*Dans le cas des effets indésirables liés aux soins, y compris les prélèvements veineux de la recherche, ils ne feront l’objet d’une transmission à l’ARS que s’ils correspondent aux critères définis dans l’arrêté du 20 février 2017 relatif aux critères de transmission à l’agence régionale de santé des signalements recueillis par les membres du réseau régional de vigilances et d’appui (<https://www.legifrance.gouv.fr/eli/arrete/2017/2/20/AFSP1705441A/jo/texte>).

Toute transmission d’effet indésirable mentionnera que le patient est inclus dans la recherche et sera tracée dans le CRF.

Dans tous les cas, la transmission pourra s’effectuer soit par le système interne de notification de l’établissement (si applicable), soit par le portail des signalements des évènements indésirables sanitaires

<https://signalement.social-sante.gouv.fr/psig_ihm_utilisateurs/index.html#/accueil> .

Ce portail retransmet la notification à la structure de vigilance concernée et adresse au déclarant un accusé de réception qui pourra être inclus dans le dossier du patient.

En cas de fait nouveau, l’investigateur, le responsable de la vigilance et le promoteur se concerteront pour discuter d’éventuelles mesures de sécurité à mettre en place, de la poursuite de l’étude et de la nécessité de contacter l’ARS.

## Modalités et durée de suivi des personnes suite à la survenue d’effets indésirables

Lorsqu’un effet indésirable persiste ou qu’un incident a des conséquences prolongées, y compris après la fin de la recherche, l’investigateur suivra la personne se prêtant à la recherche jusqu’à ce que l’évènement soit considéré comme résolu et transmettra les données de suivi à la vigilance concernée.

## Effets indésirables graves tardifs

Si l’investigateur a connaissance d’un effet indésirable survenu après la durée de suivi du patient, il le déclarera sans délai à la vigilance sanitaire concernée, selon les mêmes modalités.

# COLLECTION D'ÉCHANTILLONS BIOLOGIQUES

Dans le cadre de cette étude nous constituerons une collection biologique pour répondre aux objectifs de recherche décrits ainsi que pour réaliser d’autres analyses ou dosages au regard de faits nouveaux apportées par la littérature, la finalité de l’étude restera inchangée. Les analyses réalisées au titre de la recherche ne donneront pas lieu à un résultat diagnostique.

Les échantillons de salive recueillie (2 salivettes de 2 ml) lors des visites d’inclusion et de fin d‘étude seront stockés sur site à -20°c puis transporté dans des conditions optimales de température par l’équipe investigatrice afin d’être conservée dans les congélateurs -80°C de l’IRBA (salle dédiée, 1 place du Général Valérie André, 91223 Brétigny-sur-Orge) jusqu’à leur analyse.

Il y aura donc une collection de 1 600 salivettes (2 salivettes, 2 temps de mesures pour 400 sujets)

Le responsable des prélèvements salivaires est l’investigateur principal de l’étude.

Chaque échantillon sera identifié par un code (apposé sur des étiquettes cryo-compatibles) selon les modalités suivantes :

- Code Sujet : code Unité + numéro Sujet

- Numéro de la visite : visite d’inclusion : Vin ; pour les visites de suivi : V1, V2, V3 et V4 ; puis visite finale : Vfin. La visite additionnelle sera identifiée par le code Vadd1, Vadd2, etc en fonction de nombre de visites additionnelles réalisées.

La collection d’échantillons biologiques réalisée dans le cadre de cette recherche a été déclarée à l’autorité compétente. Après la recherche, la conservation de la collection d’échantillons biologiques sera déclarée au ministre chargé de la recherche et au directeur de l’Agence Régionale d’Hospitalisation (et soumise au CPP pour avis si changement de finalité de recherche).

# RECUEIL ET TRAITEMENT DES DONNÉES

Tout document ou objet original permettant de prouver l'existence ou l'exactitude d'une donnée ou d'un fait enregistré au cours de la recherche est défini comme document source.

Les documents sources sont constitués des CRF, des questionnaires papier ainsi que des données clinique et paraclinique recueillis sur ordinateur.

## Description des données recueillies

### Paramètres cliniques

La collecte des données cliniques se limitera aux antécédents de blessure, à l’âge, le sexe, la taille et le poids. Ces données seront enregistrées grâce au logiciel de posturographie.

### Paramètres para-cliniques

L’équilibre postural sera mesuré par un test clinique (BESS) et la posturographie.

1. Le test clinique Balance Error Scoring System (BESS)

Le test BESS évalue l’équilibre postural statique (yeux fermés) dans six conditions, en utilisant trois positions (sur les deux jambes *vs* sur une seule jambe *vs* avec les jambes en tandem) sur deux types de surface (dure *vs* souple) (Figure 5). Le participant doit rester en équilibre sans bouger pendant 20 secondes. Des pénalités sont attribuées en fonction de erreurs de posture du participant (ex : ouverture des yeux, flexion de hanche supérieure à 30°, etc.). La somme des pénalités obtenues dans les six conditions donne le score total du test BESS (Docherty et al., 2006).


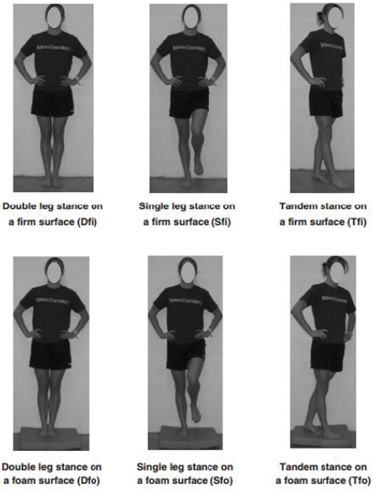


**Figure 5** - Le test clinique Balance Error Scoring System évalue l’équilibre postural statique dans six conditions, en utilisant trois positions (sur les deux jambes vs sur une seule jambe vs avec les jambes en tandem) sur deux types de surface (dure vs souple).

1. La posturographie

La posturographie est l’enregistrement de l’équilibre postural statique au moyen d’une plateforme de stabilométrie (FEETEST 6©, Techno Concept®, France) (Figure 6, ANNEXE 8). Cette dernière est constituée de quatre petites plateformes qui localisent la position du centre de pression du corps à partir des coordonnées de quatre points d’appui (talon gauche, métatarse gauche, talon droit et métatarse droit). L’enregistrement postural se fera yeux fermés et durera environ une minute.


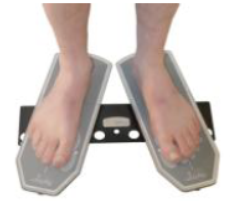


**Figure 6** – Sabots FEETEST 6© (Techno Concept®)

Afin d’assurer la reproductibilité de la mesure des paramètres para-cliniques, une formation à la réalisation du test clinique BESS sera délivrée aux investigateurs des différentes unités participant à la visite d’évaluation initiale (à l’aide d’une vidéo). De la même façon, une procédure standard sera rédigée pour la réalisation de la posturographie.

1. Données psychométriques

Des auto-questionnaires seront utilisés pour mesurer les niveaux de conscience corporelle posturale (Postural Awareness Scale) et de stress perçu (Stress perçu de Cohen).

- 1. Postural Awareness Scale (Cramer, Mehling, Saha, Dobos, & Lauche, 2018)

Ce questionnaire évalue la conscience corporelle mise en jeu dans le contrôle postural. La validation du questionnaire en français est en cours. Ce questionnaire est composé de 12 items, chaque item étant coté de 1 (ne me correspond pas du tout) à 7 (me correspond fortement). Le questionnaire évalue deux dimensions de la conscience corporelle posturale : (i) l’aisance/familiarité avec la conscience de la posture, et (ii) l’attention nécessaire pour prendre conscience de la posture.

- 1. Stress perçu de Cohen (Cohen, Kamarck, & Mermelstein, 1983)

Ce questionnaire estime la façon dont les individus évaluent leur perception du stress. Il est composé de 14 items, chaque item étant coté de 1 (jamais) à 5 (souvent). Un score élevé au questionnaire indique un haut niveau de stress perçu

Les deux questionnaires sont présentés en ANNEXE 4. Les participants rempliront les questionnaires sous format papier.

### Paramètres biologiques

Lors de la visite d’évaluation initiale et de la visite de fin d’étude, un prélèvement de salive sera effectué (2 tubes de 2 ml à chaque visite). Ces prélèvements permettront d’effectuer le suivi des marqueurs de l’axe corticotrope (cortisol et DHEA) et du système nerveux autonome (alpha-amylase et chromogranine).

## Modalités de recueil des données

Un cahier d’observation (CRF) papier sera créé pour l’étude. Celui-ci est présenté en ANNEXE 5.

Le(s) personne(s) responsables du remplissage du CRF devra/ devront être clairement identifié(s) dans le document de délégation de tâches. Le CRF sera strictement anonyme et marqué d’un numéro d’identification du participant. Pendant la recherche ou à son issue, les données recueillies et transmises au promoteur par les personnes qui dirigent et surveillent la recherche (ou tout autre intervenant spécialisé) seront rendues anonymes. Elles ne devront en aucun cas faire apparaître en clair les noms des personnes concernées ni leur adresse.

## Circuit des données

La collecte des données cliniques reposera sur une base de données cliniques conforme aux données présentes dans le CRF papier.

L’ensemble des données recueillies sur le CRF et les questionnaires seront saisies par lecture optique des documents papier (logiciel OMR Manager), les données incohérentes seront vérifiées manuellement. Ces données ainsi que les données issues de différents dosages salivaires seront concaténées sur une base de données anonyme sur un ordinateur sécurisé et à jour de logiciel de sécurité. Des sauvegardes régulières sur support amovible sécurisé seront réalisées. Ce support sera régulièrement passé en station blanche (antivirus).

L’accès à ces données sera limité aux personnes habilitées (investigateurs et collaborateurs).

La sécurité physique de l’ensemble du matériel sera assurée par la conservation dans des locaux fermés à clefs sur une enceinte militaire surveillée.

## Accès aux données et confidentialité

Le promoteur est chargé d’obtenir l’accord de l’ensemble des parties impliquées dans la recherche afin de garantir l’accès direct à tous les lieux de déroulement de la recherche, aux données sources, aux documents sources et aux rapports dans un but de contrôle de qualité et d’audit.

Les personnes qui dirigent et surveillent la recherche mettront à disposition les documents et données individuelles strictement nécessaires au suivi, au contrôle de qualité et à l’audit de la recherche, à la disposition des personnes ayant un accès à ces documents conformément aux dispositions législatives et réglementaires en vigueur.

Conformément aux dispositions législatives en vigueur notamment les articles L.1121-3 et R.5121-13 du code de la santé publique, les personnes ayant un accès direct aux données sources (par exemple, les investigateurs, les personnes chargées du contrôle de qualité, les moniteurs, les assistants de recherche clinique, les auditeurs et toutes personnes appelées à collaborer aux essais) prendront toutes les précautions nécessaires en vue d'assurer la confidentialité des informations relatives aux recherches, aux personnes qui s'y prêtent et notamment en ce qui concerne leur identité ainsi qu’aux résultats obtenus. Ces personnes, au même titre que les personnes qui dirigent et surveillent la recherche, sont soumises au secret professionnel.

Pendant la recherche ou à son issue, les données recueillies sur les personnes qui s’y prêtent et transmises au promoteur par les personnes qui dirigent et surveillent la recherche (ou tous autres intervenants spécialisés) seront codifiées. Elles ne doivent en aucun cas faire apparaître en clair les noms des personnes concernées ni leur adresse.

Les modalités de codification des participants seront les suivantes : code unité (GIGN – GI, CPA 10 – CP, 1^ier^ RPIMA – RP, 13^ième^ RDP – RD, ECOFUS – EC, BSPP – BS), numéro de sujet indiquant l’ordre d’inclusion dans l’unité, première lettre du nom et du prénom du participant seront enregistrées.

Le promoteur s’assurera que chaque personne qui se prête à la recherche a été informée sur l’accès aux données individuelles la concernant et strictement nécessaires au contrôle de qualité de la recherche.

# ASPECTS STATISTIQUES

## Calcul du nombre de sujets nécessaire

A ce jour, dans la littérature, aucune donnée sur l’efficacité des TOP dans la prévention des blessures survenant au cours de la pratique sportive n’est disponible. En revanche, les échanges avec les responsables des unités militaires et les médecins militaires suggèrent qu’une diminution du taux d’incidence de la blessure d’environ 15% serait pertinente d’un point de vue clinique. Les données épidémiologiques préliminaires des différentes unités ont montré que le taux d’incidence des blessures par chute était d’environ 50%.

Afin de déterminer la taille de l’échantillon nécessaire pour observer une différence significative du taux d’incidence entre les deux groupes, nous avons utilisé les paramètres suivants :

- test statistique : bilatéral
- taux d’incidence dans le groupe contrôle actif : 50%
- taux d’incidence dans le groupe TOP : 35%
- seuil de significativité statistique = 0,05
- puissance = 0,80

En tenant compte des potentiels perdus de vue (estimés à 10%), l’échantillon devra inclure 200 participants par groupe, soit au total 400 participants sur les deux années que durera l’étude.

## Méthodes statistiques employées

Les analyses statistiques seront réalisées avec les logiciels Matlab 2020b, R version 3.5.2 et Statistica.

Le risque d’erreur sera fixé à 5% et les tests réalisés seront bilatéraux.

Les données manquantes seront remplacées par la valeur moyenne de la variable au sein de notre échantillon pour le groupe considéré (TOP ou contrôle actif).

Tout d’abord, les effectifs des participants seront décrits dans un diagramme de flux (incluant les sujets inclus ou non inclus et les raisons de non inclusion, les sujets retenus ou non dans l’analyse et les raisons d’exclusion). Puis une analyse descriptive sera menée sur l’ensemble des variables recueillies et par groupe de comparaison (TOP *vs* contrôle actif). Les variables qualitatives seront décrites à l’aide de fréquence et de proportion, et les variables quantitatives seront décrites à l’aide de moyenne et écart-type ou médianes et intervalles interquartiles.

**Analyse de l’objectif principal**

Nous évaluerons l’effet de l’intervention (TOP *vs* contrôle actif) sur le taux d’incidence des blessures par chute au moyen d’une analyse de survie comparative (modèle de Cox, avec les variables cliniques collectées comme facteurs confondants potentiels). Les courbes de survies des groupes TOP et contrôle actif seront comparées entre elles par le test du log-rank.

**Analyse des objectifs secondaires**

Pour le premier objectif secondaire, nous évaluerons l’effet de l’intervention TOP sur le niveau de conscience corporelle posturale en comparant dans le groupe TOP *(i)* les scores au questionnaire *Postural Awareness Scale* aux six temps de mesures (ANOVA à mesures répétées), et *(ii)* les variables de la posturographie avant et après l’intervention (tests de comparaison de moyennes pour échantillons appariés).

Pour le deuxième objectif secondaire, nous évaluerons l’effet de l’intervention TOP sur l’intensité de la réponse psychobiologique de stress en comparant dans le groupe TOP aux six temps de mesure *(i)* les scores au questionnaire de stress perçu de Cohen (ANOVA à mesures répétées), et *(ii)* les variables biologiques de stress (tests de comparaison de moyennes pour échantillons appariés).

Pour le troisième objectif secondaire, nous évaluerons en machine learning la performance du modèle prédictif du risque de blessure par chute en calculant la précision de la prédiction :


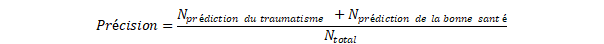


où N_prédiction du traumatisme_ est le nombre de participants chez qui le modèle de machine learning prédit la survenue d’une blessure par chute et qui, effectivement, sont victimes d’une blessure par chute au cours du stage d’évaluation ;N_prédiction de la bonne santé_ est le nombre de participants chez qui le modèle prédit la non-survenue d’un blessure par chute et qui, effectivement, ne se sont pas blessés pendant le stage d’évaluation ; et N_total_ le nombre total de participants inclus dans l’étude.

Pour le quatrième objectif secondaire, nous évaluerons l’effet de rémanence de l’efficacité de l’intervention TOP sur prévention des blessures par chute, pendant la période entre la fin de l’étude et la fin du stage d’évaluation, au moyen d’une analyse de survie comparative (modèle de Cox). Les courbes de survies des groupes TOP et contrôle actif seront comparées entre elles par le test du log-rank.

# CONTROLE ET ASSURANCE DE LA QUALITE

## Contrôle de qualité

Un attaché de recherche clinique mandaté par le promoteur visitera de façon régulière chaque structure, lors de la mise en place de la recherche, une ou plusieurs fois en cours de recherche selon le rythme des inclusions et en fin de recherche. Les éléments à revoir lors de ces visites et la fréquence de ces visites seront définis préalablement à la mise en place de l’étude en collaboration avec l’investigateur coordonnateur/principal et le responsable scientifique et selon l’évaluation du niveau de risque de l’étude.

L’étude a été identifiée comme interventionnelle à risques et contraintes minimes relevant du risque B.

Lors de ces visites, les éléments suivants seront revus :

- respect du protocole de la recherche, des procédures qui y sont définies et des textes réglementaires en vigueur,
- qualité des données recueillies dans le cahier d'observation : exactitude, données manquantes, cohérence des données avec les documents sources (dossiers médicaux, carnets de rendez-vous, originaux des résultats de laboratoire, etc,…),
- gestion des produits éventuels et des prélèvements.

Toute visite fera l’objet d’un rapport de monitorage par compte-rendu écrit.

## Audit et inspection

Un audit pourra être réalisé à tout moment par des personnes mandatées par le promoteur et indépendantes des responsables de la recherche ou une inspection conduite par les autorités de santé ou la commission nationale informatique et libertés. Il aura pour objectif de s'assurer de la qualité de la recherche, de la validité de ses résultats et du respect de la loi et des règlementations en vigueur.

Les auditeurs/inspecteurs devront avoir un accès direct aux données sources et médicales et à tout document utile lié à la conduite de l’étude clinique.

La confidentialité des données et l’anonymat des sujets seront alors respectés.

Les personnes qui dirigent et surveillent la recherche acceptent de se conformer aux exigences du promoteur et à l’autorité compétente en ce qui concerne un audit ou une inspection de la recherche.

L’audit pourra s’appliquer à tous les stades de la recherche, du développement du protocole à la publication des résultats et au classement des données utilisées ou produites dans le cadre de la recherche.

## Engagement de responsabilité

Avant de démarrer la recherche, chaque investigateur fournira au représentant du promoteur de la recherche son curriculum vitæ (CV) personnel actualisé, daté et signé. Le CV comprendra un numéro RPPS, le numéro d’inscription à l’Ordre des Médecins (hors médecins militaires) et les participations antérieures à des recherches et les formations liées à la recherche clinique.

Chaque investigateur s'engagera à respecter les obligations législatives et réglementaires et à mener la recherche conformément à la réglementation, en respectant les termes de la déclaration d'Helsinki en vigueur. L’investigateur principal de l’étude signera un engagement de responsabilités qui sera remis au représentant du promoteur.

Les investigateurs et leurs collaborateurs signeront un formulaire de délégation des tâches précisant le rôle de chacun et fourniront leur CV.

# CONSIDERATIONS ETHIQUES ET REGLEMENTAIRES

## Justification éthique du protocole

### Distinction soin / recherche

Dans le cadre du projet POSITION, les interventions TOP et contrôle actif, ainsi que l’ensemble des mesures effectuées, relèvent de la recherche.

### Rapport bénéfices / risques

#### Bénéfices

Bénéfice(s) individuel(s) :

Le projet POSITION testera l'efficacité d’une intervention TOP comme stratégie de prévention des blessures par chute au cours des activités physiques militaires. De plus, nous chercherons à décrire les facteurs de risque et de protection qui influencent la survenue d’une blessure, notamment le mécanisme cognitif de conscience corporelle posturale et la réponse psychobiologique de stress. Les résultats de ce travail doivent permettre l’élaboration de nouvelles stratégies basées sur les preuves (“evidence-based prevention/medicine”), en ce qui concerne la prédiction du risque de blessure par chute au cours des activités physiques militaires ainsi que leur prévention au moyen d’une intervention TOP. Les bénéfices attendus de ce travail concernent la protection de la santé du combattant dans le cadre de son activité professionnelle.

Bénéfice(s) collectif(s) :

A l’échelle collective, la prévention des blessures par chute permettra l’amélioration de l’état de santé de la population militaire et ainsi de sa capacité à mener les missions qui lui sont confiées. Les bénéfices de ce travail concernent à la fois la santé et la sécurité des combattants dans le cadre de leur activité opérationnelle.

#### Risques

Risque(s) individuel(s)

Aucun risque individuel en lien direct avec le protocole du projet POSITION n’est identifiable dans cette recherche.

Risque(s) collectifs(s)

Aucun risque collectif en lien direct avec le protocole du projet POSITION n’est identifiable dans cette recherche.

#### Balance bénéfice/risque

La balance bénéfice/risque est positive car l’étude comporte des bénéfices à l’échelle individuelle et collective, et aucun risque en lien direct avec le protocole du projet POSITION n’est identifié.

### Choix méthodologiques

Dans le cadre du projet POSITION, l’ensemble des mesures qui seront effectuées relèvent de la recherche et par conséquent aucune modalité supplémentaire de surveillance n’est prévue.

## Conformité aux textes de références

La recherche sera conduite conformément au présent protocole, et en accord avec la déclaration d’Helsinki (modifiée à Fortaleza en 2013 Cf. version intégrale), aux recommandations de Bonnes Pratiques Cliniques (BPC, ICHE6) ainsi qu’à toute réglementation applicable localement.

Cette recherche répondant à la définition du 2° de l'article L.1121-1 du Code de la Santé Publique, elle est soumise à l’obtention de l’avis favorable d’un Comité de Protection des Personnes (CPP).

Dans les conditions fixées par l’article L 1121-10 du code de la Santé publique, l’Etat assume l’indemnisation, pour les personnes qui s’y prêtent, des conséquences dommageables de la recherche.

Le promoteur transmettra, pour information à l’Agence nationale de sécurité du médicament et des produits de santé (ANSM), l’avis favorable du CPP et le résumé du protocole.

Les données enregistrées à l’occasion de cette recherche font l’objet d’un traitement informatisé à l’IRBA dans le respect de la loi n°78-17 du 6 janvier 1978 relative à l’informatique, aux fichiers et aux libertés modifiée par la loi 2018-493 du 20 juin 2018 et du règlement général de protection des données (articles 6.1.f, 9. *j* et l’article 13).

Cette recherche entre dans le cadre de la « Méthodologie de référence » (MR-001) en application des dispositions de l’article 73 de la loi du 6 janvier 1978 modifiée relative à l’information, aux fichiers et aux libertés. La Direction centrale du service de santé des armées, promoteur de cette étude, a signé un engagement de conformité à cette « Méthodologie de référence ».

Le responsable du traitement des données pour le Service de santé des armées est la Directrice Centrale du SSA. Le Délégué à la protection des données (DPD) est le Directeur des affaires juridiques du Ministère des Armées. Le représentant du responsable de traitement, correspondant auprès du DPD ministériel peut être contacté à l’adresse [ssa.rrt.fct@intradef.gouv.fr](mailto:ssa.rrt.fct@intradef.gouv.fr).

Cette recherche est enregistrée sur le site http://clinicaltrials.gov/ sous le n° numéro d’enregistrement.

## Amendement au protocole

Toute modification substantielle, c’est à dire toute modification de nature à avoir un impact significatif sur la protection des personnes, sur les conditions de validité et sur les résultats de la recherche, sur l’interprétation des documents scientifiques qui viennent appuyer le déroulement de la recherche ou sur les modalités de conduite de celle-ci, fait l’objet d’un amendement écrit qui est soumis au promoteur et, celui-ci doit obtenir, préalablement à sa mise en œuvre, un avis favorable du CPP.

Les modifications non substantielles, c'est-à-dire celles n’ayant pas d’impact significatif sur quelque aspect de la recherche que ce soit, sont communiquées au CPP à titre d’information.

Tous les amendements au protocole doivent être portés à la connaissance de tous les professionnels de santé qui participent à la recherche et qui s’engagent à en respecter le contenu.

# CONSERVATION DES DOCUMENTS ET DES DONNEES RELATIVES A LA RECHERCHE

Les documents suivants relatifs à cette recherche seront archivés conformément aux Bonnes Pratiques Cliniques **pour une durée de 15 ans** suivant la fin de la recherche :

- Par les investigateurs :
- Le protocole et les amendements éventuels au protocole,
- Un exemplaire des consentements éclairés signés des participants,
- Les cahiers d’observation,
- Les dossiers sources des participants,
- Tous les autres documents et courriers relatifs à la recherche.

Tous ces documents sont sous la responsabilité du médecin pendant la durée réglementaire d’archivage.

- Par le promoteur :
- Le protocole et les amendements éventuels au protocole,
- Un exemplaire des consentements éclairés signés des participants,
- L’original des cahiers d’observation,
- Tous les autres documents et courriers relatifs à la recherche.

Tous ces documents sont sous la responsabilité du promoteur pendant la durée réglementaire d’archivage.

Aucun déplacement ou destruction ne pourra être effectué sans l’accord du promoteur. Au terme de la durée réglementaire d’archivage, le promoteur sera consulté pour destruction et donnera son accord écrit. Toutes les données, tous les documents et rapports pourront faire l’objet d’audit ou d’inspection.

# REGLES RELATIVES A LA PUBLICATION

## Communications scientifiques

L’analyse des données sera réalisée par l’IRBA. Cette analyse donnera lieu à un rapport écrit qui est soumis à la Direction centrale du Service de santé des armées. Ce rapport permettra la préparation d’une ou plusieurs publication(s).

Toute communication écrite ou orale des résultats de la recherche devra recevoir l’accord préalable de la personne qui dirige et surveille la recherche et, le cas échéant, de tout comité constitué pour la recherche.

La publication des résultats principaux mentionnera le nom du promoteur (Direction centrale du Service de santé des armées), de tous les professionnels de santé ayant inclus ou suivi des patients dans la recherche, des méthodologistes, biostatisticiens et data managers ayant participé à la recherche et des membres du(des) comité(s) constitué(s) pour la recherche. Il sera tenu compte des règles internationales d’écriture et de publication (Convention de Vancouver, février 2006 ; Recommendations for the Conduct, Reporting, Editing, and Publication of Scholarly Work in Medical Journals Updated, IJCME, 2018).

## Communication des résultats aux patients

Conformément à l'article L1122-1 du code de la santé publique, les patients seront informés des résultats globaux de la recherche par sous la forme d’une présentation orale effectuée dans les différentes unités militaires.

## Cession des données

Le recueil et la gestion des données seront assurés par l’IRBA. Les conditions de cession de tout ou partie de la base de données de la recherche seront décidées par la Direction centrale du Service de santé des armées (promoteur de la recherche) et feront l’objet d’un contrat écrit.

# REFERENCES

Amboni, M., Barone, P., & Hausdorff, J. M. (2013). Cognitive contributions to gait and falls: evidence and implications. *Movement disorders, 28*(11), 1520-1533.

At, W. (2016). *Epidémiologie de la traumatologie aigue sportive chez les sapeurs-pompiers en phase d'incorporation.* (Thèse de médecine générale), Université Paris 13, Paris.

Bahr, R., & Holme, I. (2003). Risk factors for sports injuries--a methodological approach. *British Journal of Sports Medicine, 37*(5), 384-392. doi:10.1136/bjsm.37.5.384

Bauvent, Y. (2014). *Etude de la tolérance et de l’état de fatigue au cours de la préparation physique initiale des EVAT au CFIM de Gap. Etude de l’effet sur les capacités en endurance et en force.* (Thèse de médecine générale), Université Aix Marseille, Marseille.

Bertrand, M. (2016). *Pathologies traumatiques au cours du stage commando marine : analyse des interactions entre vigilance et proprioception.* (Thèse de médecine générale), Université Paris XI, Paris.

Bigard, X., Cravic, J. Y., & Banzet, S. (2010). Prévention des risques liés à la préparation physique du militaire: synthèse des connaissances actuelles. *Médecine et Armées, 38*(1), 07-16.

Brocard, M. (2014). *Pathologies traumatiques au bataillon des Marins Pompiers de Marseille, étude prospective de juin 2012 à avril 2013.* (Thèse de médecine générale), Université Aix Marseille, Marseille.

Chipault, M. (2016). *Epidémiologie de la pratique sportive et des blessures liées aux activités physiques et sportives chez les personnels naviguants militaires français.* (Thèse de médecine générale), Université Paris-Sud, Paris.

Cohen, S., Kamarck, T., & Mermelstein, R. (1983). A global measure of perceived stress. *Journal of health and social behavior*, 385-396.

Craig, A. D. (2002). How do you feel? Interoception: the sense of the physiological condition of the body. . *Neuroscience, 3*(8), 655-666.

Cramer, H., Mehling, W. E., Saha, F. J., Dobos, G., & Lauche, R. (2018). Postural awareness and its relation to pain: validation of an innovative instrument measuring awareness of body posture in patients with chronic pain. *BMC musculoskeletal disorders, 19*(1), 109.

Crosnier, S. N. (2013). *Évaluation du sommeil des sous-mariniers en situation opérationnelle sur Sous-marins Nucléaires Lanceurs d’Engins : Influence des Techniques d’Optimisation du Potentiel sur le sommeil. .* (Doctorat en médecine).

Delahunt, E., Bleakley, C. M., Bossard, D. S., Caulfield, B. M., Docherty, C. L., Doherty, C., . . . Gribble, P. A. (2018). Clinical assessment of acute lateral ankle sprain injuries (ROAST): 2019 consensus statement and recommendations of the International Ankle Consortium. *British Journal of Sports Medicine, 52*(20), 1304-1310. doi:10.1136/bjsports-2017-098885

Dirand, E. (2014). *Préparation mentale : réinstaurons l’indispensable duo « santé-performances » de nos soldats*. (Mémoire professionnel de M2 STAPS, spécialité EMIS).

Docherty, C. L., Valovich McLeod, T. C., & Shultz, S. J. (2006). Postural control deficits in participants with functional ankle instability as measured by the balance error scoring system. *Clin J Sport Med, 16*(3), 203-208.

Etat-major des armées - Centre national des sports de la défense. (2011). *Manuel d’Entrainement Physique Militaire et Sportif*.

Forbes, P. A., Chen, A., & Blouin, J. S. (2018). Sensorimotor control of standing balance *Handbook of Clinical Neurology* (Vol. 159, pp. 61-83): Elsevier.

Gage, W. H., Winter, D. A., Frank, J. S., & Adkin, A. L. (2004). Kinematic and kinetic validity of the inverted pendulum model in quiet standing. *Gait & Posture, 19*(2), 124-132.

Longin, A. (2015). *Mécanismes de survenue des traumatismes des membres inférieurs au cours du stage commando marine.* (Thèse de médecine générale), Université Brest - Bretagne Occidentale, Brest.

Meeuwisse, W. H. (1994). Assessing causation in sport injury: a multifactorial model. *Clin J Sport Med, 4*, 166–170.

Mehling, W. E., Gopisetty, V., Daubenmier, J. J., Price, C. J., Hecht, F. M., & Stewart, A. (2009). Body awareness: construct and self-report measures. *PLoS One, 4*(5), e5614.

Millet, C. (2013). *Influence des Techniques d’Optimisation du Potentiel sur la préparation mentale à l’éjection chez des pilotes.* Mémoire de diplôme universitaire.

Morinière, N. (2013). *Etude prospective des pathologies médicales et traumatiques au cours du stage commando marine et proposition de mesures de prévention.* (Thèse de médecine générale), Université Brest-Bretagne occidentale, Brest.

Pagninia, F., Manzonib, G. M., Castelnuovoab, G., & Molinariab, E. B. (2013). A brief literature review about relaxation therapy and anxiety. *Body, Movement and Dance in Psychotherapy, 8*(2), 71-81.

Pleche, S. (2018). *Étude des pathologies sportives en lien avec la formation militaire initiale chez des jeunes engagés du Centre de Formation des Militaires du rang d’Angoulême, et engagés sous-officiers de l’École de Saint-Maixent.* (Thèse pour le doctorat en médecine), Université de Bordeaux, Bordeaux.

Ressort, T., Desjeux, G., Marsan, P., & Thevenin-Garron, V. (2013). Les affections en service liées aux sports chez les militaires français. *Santé publique, 25*(3), 263-270.

Steiler D., D. J., Trousselard M. . (2011). *Developing positive emotions for the improvement of first year students’ well-being*. Paper presented at the International Congress of Positive Psychology, Philadelphie, USA.

Trousselard, M., Fidier, N., Ferhani, O., & Perraut-Pierre, E. (2010). *Détermination d’un outil de mesure de l’impact des stress vécus en fonction de la résilience des individus : intérêt pour l’évaluation de l’efficacité des mesures de prévention.*, (Rapport de synthèse intermédiaire du contrat d’objectif 10co708, I. Brétigny-sur-Orge).

Trousselard M., P. E., Ferres Saint Aubin K. (2010). *Impact of positive emotions enhacement on physiological processes and psychological functioning in military pilots.* . Paper presented at the Human factor & medicine panel symposium (HFM), , Sofia, Bulgaria.

Vealey, R. S., & Greenleaf, C. A. I. (2010). Seeing in believing: understanding and using imagery in sport. In J. M. Williams (Ed.), *Applied sport psychology: personal growth to peak performance* (pp. 267-304).

Verdonk, C., Duffaud, A., Longin, A., Bertrand, M., Canini, F., & Trousselard, M. (2019). *Equilibre postural et risque de blessure au stage commando marine*. Paper presented at the XIVème Journée des internes et des assistants, Ecole du Val-de-Grâce (Paris).

Verdonk, C., Trousselard, M., Medani, T., Vialatte, F., & Dreyfus, G. (2020). Probing the posture with machine learning supports the enhanced body awareness hypothesis in trait mindfulness. *(submitted)*.

Volchan, E., Rocha-Rego, V., Bastos, A. F., Oliveira, J. M., Franklin, C., Gleiser, S., . . . Figueira, I. (2017). Immobility reactions under threat: A contribution to human defensive cascade and PTSD. *Neurosci Biobehav Rev, 76*(Pt A), 29-38. doi:10.1016/j.neubiorev.2017.01.025

William, J. M., Zinsser, N., & Bunker, L. (2010). Cognitive techniques for building confidence and enhancing performance. In J. M. William (Ed.), *Applied sport psychology: personal growth to peak performance* (pp. 306-355).

Williams, J. G. P. (1971). A etiological classification of injuries in sportsmen. *British Journal of Sports Medicine, 5*(4), 228-230.

World Health Organization. (2003). *Health and development through physical activity and sport*. Retrieved from

# ANNEXES

**ANNEXE 1**- Résumé synthétique des études ayant évalué les effets d’une formation TOP sur les performances psycho-cognitives et physiques, sur le stress et sur le sommeil.

| **Population** | **Performance / Objectif visés** | **Résultats / Limites** | **Référence** | **Programme TOP** |
| --- | --- | --- | --- | --- |
| Etudiants en 1^ière^ année de médecine (élève officier) et d’école de commerce | Performances cognitives | Bénéfices des T.O.P. sur le sentiment d’efficacité pour la préparation à un concours/examen  Limites : absence de contrôle | (Steiler D., 2011) | 8 semaines  1h/semaine + exercices de pratiques quotidiens |
| Pilotes de l’armée de l’air | Performances cognitives | Bénéfice des T.O.P. sur la prise de décision d’éjection en simulateur  Limite : 5 sujets | (Millet, 2013) | Mémoire non consultable |
| 16^ième^ BC en mission Sentinelle | Performances psycho-cognitive et physiques, niveau de stress | Projet PHYTOP  Bénéfices sur les performances cognitives (test mnésique) et amélioration des ressources qui permettent la tolérance au stress (optimisme et motivation) | Non publié | Entrainement 1h30 hebdomadaire associée à 1h30 de pratique sportive encadrée - 9 semaines |
| Brigade de Sapeurs-Pompiers de Paris – BSPP | Gestion du stress  Etude de l’efficacité des T.O.P. et de la cohérence cardiaque (CC) sur des variables psychopathologiques | Efficacité des techniques sur le stress perçu, l’humeur négative, l’immunité locale et la mesure de stress post-traumatique.  La CC semble plus efficace sur l’immunité alors que les T.O.P. diminuent le stress perçu de façon plus efficace.  Etude randomisée monocentrique | (Trousselard, Fidier, Ferhani, & Perraut-Pierre, 2010) | Entrainement régulier pendant 2 mois |
| Sous-mariniers en situation opérationnelle en Sous-marins Nucléaires Lanceurs d’Engins | Gestion du stress /Sommeil  Association T.O.P et CC | Bénéfices des T.O.P. à la fois les sous-mariniers de quart et les sous-mariniers de hors-quart.  Evaluation mixe : subjective pour les troubles de l’humeur et objective pour la mesure du sommeil en conditions opérationnelles | (Crosnier, 2013) | Mémoire non consultable |
| Pilotes de l’armée de l’air en OPEX, Afghanistan | Performance opérationnelle et stress perçu en mission | Diminution du stress perçu, du sentiment de fatigue, de l’excrétion urinaire de cortisol, ainsi qu’une amélioration de la cohésion du groupe, notamment dans la cohésion au chef d’escadron (cohésion verticale) pour les deux escadrons ayant bénéficié de ces techniques.  Etude randomisée | (Trousselard M., 2010) | 8 semaines  1h/semaine + exercices de pratiques entre 2 séances |
| Militaires d’infanterie | Performance opérationnelle | Bénéfices d’une formation courte et intense aux T.O.P (deux heures quotidiennes) sur les performances motrices (montée de cordes) et techniques (montage et démontage du FAMAS) | (Dirand, 2014) | 2 heures quotidiennes – sur une courte période |

**ANNEXE 2** - Fiche type de déroulé d’une séance TOP et descriptif du contenu des séances


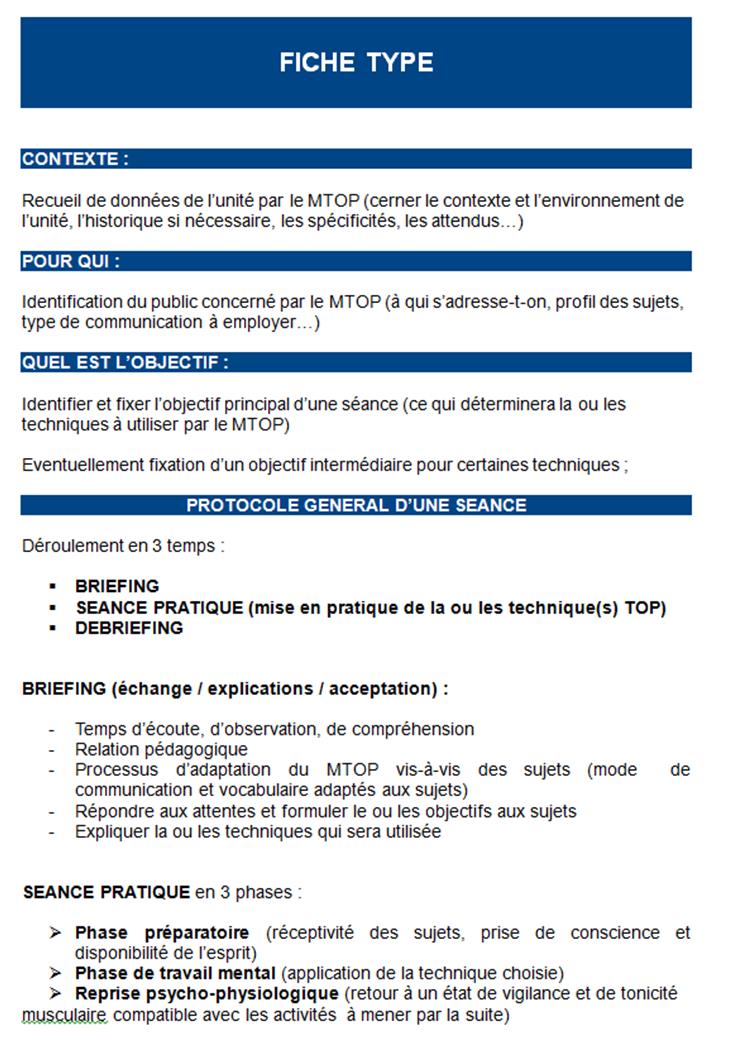


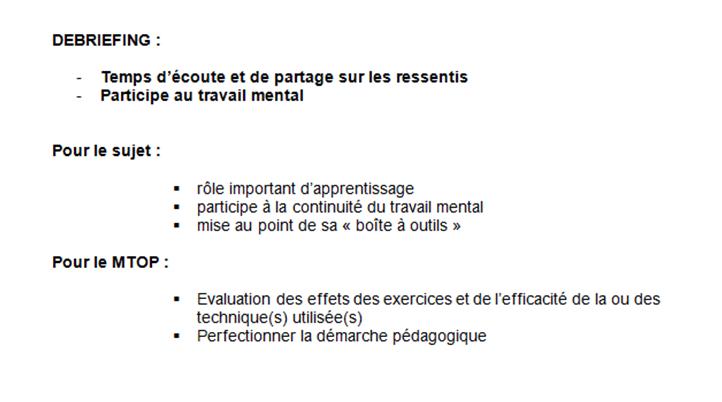


**ANNEXE 2** - descriptif du contenu des séances TOP

- **Objectif général :** optimiser la conscience corporelle
- **Objectifs intermédiaires** :
- Prise de conscience corporelle
- Conscientiser les différentes parties du corps

**Intervenants**

1 à 2 Moniteurs TOP (par séance et par unité (à définir)

**Programmes des 6 séances TOP**

**Séance 1 : Présentation des TOP, du déroulement des séances et des objectifs - 1ers exercices**

1. **Prise de conscience** de la **respiration**

-> Prise de conscience des sensations

- - - Moyens : initiation aux TOP par différents *exercices de respirations*

1. Amorçage de la « boîte à outils » TOP

**Séance 2 : Techniques de relaxation**

1. **Poursuite de la conscientisation** sur l’ensemble du corps par la **relaxation** -récupération et la détente
   - - Moyens : *techniques de relaxation* : choix d’une ou plusieurs techniques appropriées au contexte et aux participants

Reprise psycho-physiologique avant reprise des activités.

- la relaxation se fonde sur la constatation que le **tonus musculaire** est en étroite relation avec les émotions, l’affectif et le mental.

- la relaxation conduisant à un **état d’hypovigilance** favorisant la récupération

- la détente permettant d’évoluer vers **l’état de vigilance** soit pour effectuer un travail d’imagerie mentale soit pour mener à bien une activité stressante

**Séance 3 : Approfondissement d’une technique de relaxation basée sur la posture**

1. Approfondissement de la conscientisation corporelle (séance 1 et 2 mises à profit)
   - - Moyen : *technique de Relaxation Posturale* (RPo) impliquant les muscles à empreintes « émotionnelles »

- Méthode mise au point grâce aux retours « d’expérience » des missions PAMIR et SERVAL : en cause les fortes sollicitations psychologiques et musculaires des militaires mettant en évidence la relation entre le mental et le physique.

**Séance 4 : séance de Dynamisation Psycho-Physiologique (DPP)**

1. Mise en condition avant une activité à composante physique
   - - Moyen : *technique de Dynamisation Psycho-physiologique (DPP)*

- elle favorise la motivation et initie à la préparation mentale

- mouvements qui se rapprocheront de l’activité qui suivra (si possible) pour augmenter son efficacité (recueil de données en amont de la séance)

**Séance 5 : Séance de Projection Mentale de la Réussite (PMR)**

1. Aborder positivement et en pleine possession de ses moyens une situation
   - - Moyen : *technique de Projection Mentale de la Réussite (PMR)*

- Technique essentiellement de dynamisation qui agit sur la représentation et la perception que le sujet a de lui-même et de la situation à laquelle il sera confronté.

- favorise l’équilibrage ou le rééquilibrage de la balance des perceptions

**Séance 6 : Séance de Pré-activation Mentale (P-AM) ou PMR partielle**

1. Favoriser l’évolution vers le niveau d’activation optimal (zone de stress adapté) pour préparer une activité
   - - Moyen : *technique de Pré-activation Mentale (P-AM) ou PMR partielle*

- Technique de dynamisation mentale et d’anticipation

- Elle optimise et rend « opérant » tout le travail technique, intellectuel, physique et mental préalable.

La mise au point de la « boîte à outils » personnelle des TOP

passant par le protocole d’enseignement rigoureux **F.E.A** : **F**ormation / **E**ntraînement / **A**pplication

**ANNEXE 3** - Détails des séances d’activités cognitives du groupe Contrôle actif

| **N° Séance** | **Fil conducteur** | **Processus cognitif travaillé** | **Matériel** | **Activité(s) proposée(s)** |
| --- | --- | --- | --- | --- |
| 1 | Présentation et identification des différents canaux sensoriels (visuel, auditif, olfactif et gustatif) | Perception et attention | Salle, matériel informatique | *Exercice narratif d’une expérience positive / négative  *Filtre attentionnel : vidéos *Exercices : jeu des 7 différences, détection des visages dans une image |
| 2 | Raisonnement déductif et logique sous contrainte de temps | Raisonnement | Sur papier plastifié | Exercice type "psychotechniques"  (ressource : livres d’entraînement aux concours d’entrée) |
| 3 | Intégration des informations | Mémorisation | - | Exercice permettant un réinvestissement : techniques de mémorisation, cartes mentales  (stratégie : Palais des mémoires)  Eléments à mémoriser (code, message sous contrainte, liste de course, etc.) |
| 4 | Conceptualisation, expression et transmission d'idées | Dissertation | - | Enoncés de culture générale (histoire du régiment, thématique choisi par le participant, sujet d’actualité, etc.) |
| 5 | Procédure mentale avant action | Imagerie mentale, perception, action | - | Ateliers : montage / démontage armes, nœuds marins, tangrammes, |
| 6 | Créativité | Imagination, pensée divergente et convergente* | - | Exercices de « pensée active » / batterie d’efficience créative : exercices de résolution de problèmes (ex : aller d’un point A à un point B par tous les chemins possibles) |

* Pensée divergente : produire de nombreuses idées à partir d’un stimulus et pensée convergente : produire une seule solution intégrant plusieurs éléments

**ANNEXE 4** - Questionnaires PAS (Postural Awareness Scale) et de Cohen (stress perçu)

| **Questionnaire PAS** | | 1  Ne me correspond pas du tout | 2 | 3 | 4  Me correspond moyennement | 5 | 6 | 7  Me correspond fortement |
| --- | --- | --- | --- | --- | --- | --- | --- | --- |
| 1 | J'ai besoin d'être très concentré(e) pour prendre conscience de ma posture corporelle |  |  |  |  |  |  |  |
| 2 | Quand je me tiens dans une mauvaise posture corporelle, souvent je ne la remarque pas avant qu'elle devienne douloureuse |  |  |  |  |  |  |  |
| 3 | Quand je suis assis(e), j’ai souvent tendance à m’avachir |  |  |  |  |  |  |  |
| 4 | Quand je me concentre sur une activité spécifique, je prends souvent une posture corporelle particulière sans m'en rendre compte |  |  |  |  |  |  |  |
| 5 | Quand je me concentre sur une activité spécifique, je prends souvent une posture corporelle particulière sans m'en rendre compte |  |  |  |  |  |  |  |
| 6 | Quand je me concentre sur une activité spécifique, je prends souvent une posture corporelle particulière sans m'en rendre compte |  |  |  |  |  |  |  |
| 7 | Au travers de ma posture corporelle, je peux intentionnellement modifier l'impression que je donne aux autres |  |  |  |  |  |  |  |
| 8 | Tout au long de la journée, je suis en permanence conscient(e) de la façon dont je suis assis(e) ou debout |  |  |  |  |  |  |  |
| 9 | Je suis souvent conscient(e) / me rends compte de ma posture corporelle du moment, que je sois assis(e) ou debout |  |  |  |  |  |  |  |
| 10 | Même si je suis focalisé(e) sur quelque chose, je suis en permanence conscient(e) de ma posture corporelle |  |  |  |  |  |  |  |
| 11 | Au travers de ma posture corporelle, je peux contrôler consciemment mon humeur |  |  |  |  |  |  |  |
| 12 | Je remarque si ma posture corporelle est bonne pour moi, ou non, seulement quand je me concentre dessus |  |  |  |  |  |  |  |

**Questionnaire de Stress perçu de Cohen**

**
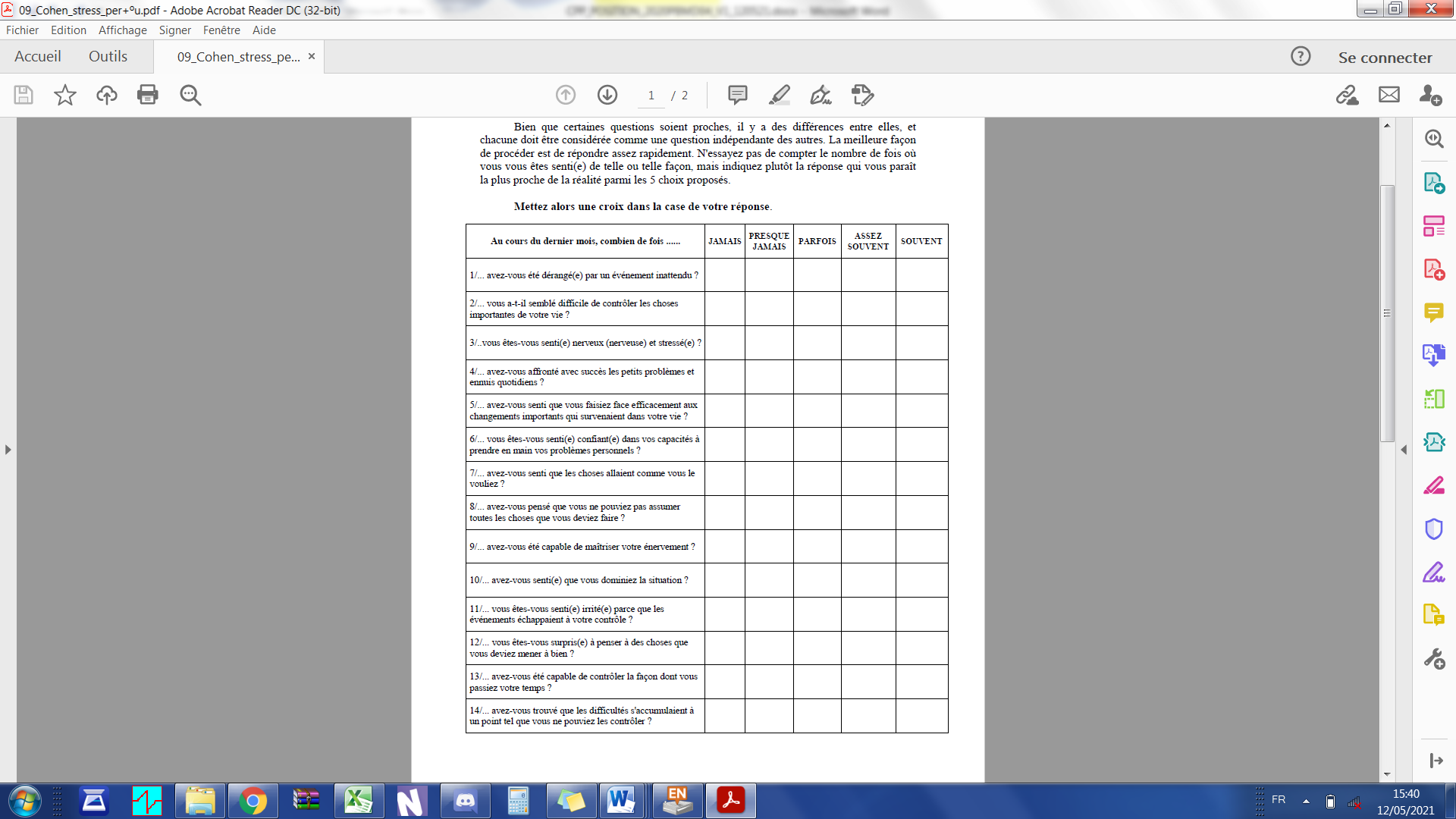
**

1. Dans la suite du document, par souci de lisibilité, nous utiliserons le terme « blessure par chute » pour désigner une blessure (ou un traumatisme) par chute faisant suite à un déséquilibre. [↑](#footnote-ref-1)
2. Une formation « avancée » aux TOP est définie comme une formation dont la durée est supérieure à celle de la formation initiale proposée par le Ministère des armées (10 heures). [↑](#footnote-ref-2)
3. Toute candidate féminine doit présenter un test de grossesse négatif pour participer au stage de sélection. [↑](#footnote-ref-3)
